# Diffusible signal factor (DSF)-mediated quorum sensing modulates expression of diverse traits in *Xanthomonas citri* and responses of citrus plants to promote disease

**DOI:** 10.1186/s12864-018-5384-4

**Published:** 2019-01-17

**Authors:** Lei Li, Jinyun Li, Yunzeng Zhang, Nian Wang

**Affiliations:** 1 Chinese Academy of Agricultural Sciences, Institute of Vegetables and Flowers, Beijing, 100081 China; 2 Citrus Research and Education Center, Department of Microbiology and Cell Science, University of Florida, Lake Alfred, FL 33850 USA

**Keywords:** *Xanthomonas*, Diffusible signal factor (DSF), Quorum sensing, Citrus canker, Transcriptomic profiling

## Abstract

**Background:**

The gram-negative *Xanthomonas* genus contains a large group of economically important plant pathogens, which cause severe diseases on many crops worldwide. The diffusible signal factor (DSF) - mediated quorum sensing (QS) system coordinates expression of virulence factors in plant pathogenic *Xanthomonas* spp. However, the regulatory effects of this system during the *Xanthomonas-* plant interactions remain unclear from both the pathogen and host aspects.

**Results:**

In this study, we investigated the *in planta* DSF- mediated QS regulon of *X. citri* subsp*. citri* (*Xac*), the causal agent of citrus canker. We also characterized the transcriptional responses of citrus plants to DSF-mediated *Xac* infection via comparing the gene expression patterns of citrus trigged by wild type *Xac* strain 306 with those trigged by its DSF- deficient (∆*rpfF*) mutant using the dual RNA-seq approach. Comparative global transcript profiles of *Xac* strain 306 and the ∆*rpfF* mutant during host infection revealed that DSF- mediated QS specifically modulates bacterial adaptation, nutrition uptake and metabolisms, stress tolerance, virulence, and signal transduction to favor host infection. The transcriptional responses of citrus to DSF-mediated *Xac* infection are characterized by downregulation of photosynthesis genes and plant defense related genes, suggesting photosynthetically inactive reactions and repression of defense responses. Alterations of phytohormone metabolism and signaling pathways were also triggered by DSF-mediated *Xac* infection to benefit the pathogen.

**Conclusions:**

Collectively, our findings provide new insight into the DSF- mediated QS regulation during plant-pathogen interactions and advance the understanding of traits used by *Xanthomonas* to promote infection on host plants.

**Electronic supplementary material:**

The online version of this article (10.1186/s12864-018-5384-4) contains supplementary material, which is available to authorized users.

## Background

The genus *Xanthomonas* comprises a large group of gram-negative plant pathogenic bacteria that have considerable agricultural impact worldwide, and therefore, is an important model genus for studying the host-pathogen interactions [1, 2]. Successful infection and bacterial multiplication of *Xanthomonas* spp. in host tissues require coordinated expression of a combination of virulence factors. Key virulence factors of *Xanthomonas* spp. include, among others, the type III secretion system (T3SS) and its effectors [3, 4], bacterial polysaccharides such as the xanthan extracellular polysaccharides (EPS) and lipopolysaccharides (LPS) [5], and cell wall degrading enzymes [1]. Expression of these virulence factors is regulated by different extracellular stimuli via multiple coordinated regulatory systems, including cell-to-cell communication (quorum-sensing, QS) pathways, two-component systems and various transcriptional regulators [1].

The QS regulatory systems of *Xanthomonas* are mediated by molecules belonging to the diffusible signal factor (DSF) family [2, 6, 7]. The DSF-mediated QS has been studied most extensively in the crucifer pathogen *X. campestris* pv. *campestris* (*Xcc*). The synthesis and perception of the DSF signal, which was identified as *cis*-11-methyl-2-dodecenoic acid [8], require the *rpf* gene cluster (for regulation of pathogenicity factors), including *rpfB, rpfF* and *rpfGHC* [9, 10]. RpfB was initially thought to be involved in DSF biosynthesis, but it was later identified as a fatty Acyl-CoA ligase involved in the turnover of the DSF family of signals in *Xanthomonas* [11]. The RpfF protein, functioning as a putative enoyl-CoA hydratase, is responsible for the synthesis of DSF. RpfC and RpfG consist of a two-component system involved in DSF perception and signal transduction. RpfC is a hybrid sensor kinase and RpfG is a response regulator with a CheY-like receiver (REC) domain and an HD-GYP domain, capable of degrading the second messenger cyclic di-GMP [6, 10, 12, 13]. DSF can bind directly to the N-terminal, 22 amino acid-length sensor region of RpfC and activate RpfC autokinase activity to regulate QS and virulence in *Xcc* [14]. RpfH is a putative membrane protein with no known role in DSF signaling [10].

The contribution of DSF/Rpf regulatory system to virulence has been demonstrated in many members of Xanthomonas. For example, DSF signaling in *Xcc* influences the synthesis of a range of virulence factors including extracellular enzymes such as endoglucanase, protease, and endomannanase, and the xanthan EPS, as well as alterations in biofilm formation [6, 10, 15]. Specifically, the RpfS- dependent second DSF signaling pathway controls expression of genes involved in type IV secretion and chemotaxis and therefore affects bacterial motility, suggesting a role in the epiphytic phase of the *Xcc* disease cycle [16]. Similarly, the DSF-mediated QS has been shown involved in early attachment and *in planta* growth of *Xac* in the citrus host during the citrus canker disease cycle [17]. Recent report indicates that the DSF family in *Xcc* elicited plant innate immunity and this effect was suppressed through the secretion of the xanthan exopolysaccharide [18]. DSF also confers a fitness advantage to *Xcc* during interspecies competition [19].

Transcriptome profile, functional genomics, and proteomic analyses have significantly advanced the understanding of the DSF/Rpf regulatory network and its role in pathogenesis of *Xanthomonas. Earlier studies have revealed that the* RpfC/RpfG two-component system coordinately regulates the expression of various genes related to virulence via the cyclic di-GMP signaling that activates the transcriptional activators Clp and Zur *in Xcc* [6, 12, 13, 20]. These include the genes encoding extracellular enzymes, components of type II secretion system (T2SS), components of type III secretion system (T3SS), and the genes involved in EPS production. Comparative transcriptome studies using whole-genome microarray showed that the DSF/Rpf -mediated QS regulation in the citrus canker pathogen *X. citri* subsp*. citri* (*Xac*) is growth phase-dependent, and more genes in the exponential phase are differentially regulated by the RpfC/RpfG system compared with in the stationary phase [17]. The RpfC/RpfG system-regulated genes include diverse genes involved in chemotaxis and motility, flagellar biosynthesis, production of extracellular enzymes and adhesins, stress tolerance, regulation, transport, and detoxification [17]. There are also some unique genes controlled by RpfF, RpfC or RpfG alone, indicating the complexity of the QS pathway and the involvement of additional DSF signal perception and transduction mechanisms in *Xac* [17]. Interestingly, recent studies suggested additional signaling outputs from RpfC and an interaction of RpfG with a second unknown sensor [16, 21]. The authors found that DSF and RpfC also regulate expression of a number of genes encoding transcriptional regulator, hydrolase, protease and hypothetical proteins independently of RpfG, and RpfG regulates expression of genes involved in chemotaxis, signal transduction and protein export, independently of RpfF or RpfC [16, 21]. These studies also revealed that RpfC can recognize other unidentified environmental signals (in addition to DSF) [21] and the DSF signal can be recognized by a second sensor RpfS, a PAS domain-containing histidine kinase that regulates genes involved in type IV secretion and chemotaxis in a pathway independent of RpfC and RpfG [16]. Our knowledge of the protein(s)/regulator(s) acting downstream of RpfS in DSF signal transduction cascades remains limited. In addition, the DSF/Rpf system controls three non-coding RNA (ncRNAs) that contribute to virulence in *Xcc* [21].

Comparative proteomic analysis revealed diverse regulatory effects of DSF/Rpf in *Xcc* on proteins involved in regulation, biosynthesis and intermediary metabolism, stress tolerance, and motility [22]. Similarly, mutation of the *rpfF* gene has a substantial impact on the proteome of *X. oryzae* pv. *oryzicola*, affecting proteins involved in a range of functions including nitrogen transfer, protein folding, resistance to oxidative stress and flagellar synthesis [23]. Interestingly, for many of the proteins regulated by the DSF/Rpf system in *Xcc*, the alteration in abundance was not associated with alteration in transcript level, suggesting that both posttranscriptional regulation and post-translational turnover may occur [22].

Despite the extensive transcriptome analyses of the DSF/Rpf regulatory system in Xanthomonas spp. as stated above, most of which were performed using the bacterium grown under culture media conditions, and knowledge on regulatory effects of the DSF/Rpf system of Xanthomonas spp. during the interaction with host plants is still lacking. The actions of the elements involved in DSF signaling and the role of DSF signal transduction during plant infection remain to be determined from both the pathogen and host aspects. In the present study, we investigated the DSF/Rpf QS regulation *in planta* during *Xac* infection of citrus host. Metatranscriptome analysis of the compatible interaction between *Xac* and citrus was conducted using RNA-Seq to compare the global transcriptomes of wild-type and isogenic *rpfF* mutant (∆*rpfF*) strains of Xac, as well as the citrus transcriptional patterns in response to their infection. This work provides a comprehensive picture of the genes and traits regulated by the DSF/Rpf QS in *Xac in planta* and host responses to the DSF-mediated infection.

## Methods

### Bacterial strains and growth conditions

*Xanthomonas citri* subsp. *citri* (*Xac*) wild type strain 306 [24] and its *rpfF* gene deletion (∆*rpfF*) mutant strain [17] were grown at 28 °C with shaking at 200 rpm. in nutrient broth (NB; Difco, Detroit, MI) containing rifamycin (50 μg/mL). Bacterial growth was measured in a spectrophotometer at 600 nm.

### Plant inoculations and sampling of infected leaves

Plant inoculation was performed as described in our previous work [5]. Briefly, young (about 12-week-old) Duncan grapefruit (*Citrus paradise* Macfadyen) plants were grown in potting medium/soil in a greenhouse at the Citrus Research and Education Center, Lake Alfred, FL, USA, and maintained at approximately 25–30 °C and a 55% relative humidity until the primary leaves were fully expanded but not fully matured. The bacterial inoculum cells were grown as described above. When the cells reached late-log phase (OD_600_ = 1.0; 5 × 10^10^ cfu/mL), they were collected by centrifugation at 4000 rpm for 15 min. The cell pellets were resuspended in sterilized 0.85% NaCl, washed, and resuspended in sterilized 0.85% NaCl to a final density of 5 × 10^6^ cfu/mL. To establish in planta populations, bacteria were introduced by infiltration into leaves using a needleless syringe. Infiltrated plants were maintained in the same greenhouse for canker symptom development. All plant inoculations included at least three leaves at a similar developmental stage from each plant, and ten replicate plants were inoculated for each strain. Time- course bacterial growth *in planta* was tested as described previously [5]. All the tests were repeated three times. Based on the progression of development of canker symptoms, the infected leaves were sampled at 5 days post inoculation (DPI) for RNA extraction. The inoculated leaf area was collected using a cork borer (leaf area, 1 cm^2^) and 10 leaf samples from each biological replicate (three replicates for each treatment) were pooled and immediately frozen in liquid nitrogen, and kept in − 80 °C until process for RNA isolation.

### RNA extraction, library construction and Illumina RNA-seq

Total RNA was extracted from leaf samples using RNeasy Plant Mini Kit (Qiagen, Valencia, CA) and contaminated DNA was removed by treatment with RNase-Free DNase Set (Qiagen, Valencia, CA). The quality and quantity of RNA samples were assessed using NanoDrop ND-1000 spectrophotometer (NanoDrop Technologies, Wilmington, DE), Agilent 2100 Bioanalyzer (Agilent Technologies, Santa Clara, CA) and agarose gel electrophoresis. The total RNA was treated with DNase I (New England Biolabs, Ipswich, MA) prior to library construction. The rRNA of plant and bacteria was depleted using Ribo-Zero™ rRNA Removal Kits (Plant Leaf) and Ribo-Zero™ rRNA Removal Kits (Bacteria) respectively, according to the manufacturer’s instructions (Epicenter Technologies, Madison, WI). Poly (A) + mRNA was purified using Agencourt RNAClean XP Kit (Beckman Coulter Life Sciences, Indianapolis, IN) and fragmented into short pieces. Using these short fragments as templates, first strand cDNA synthesis was conducted using random hexamer-primers and SuperScript® II Reverse Transcriptase (Invitrogen, Waltham, MA), and the second-strand cDNA was synthesized using RNase H (Invitrogen, Waltham, MA) and DNA polymerase I (New England Biolabs, Ipswich, MA). After purification, end repair, and ploy (A) tails add, the cDNA fragments were ligated to sequencing adapters. Then fragments of an appropriate size were purified and amplified by PCR to produce the final library. Finally, the cDNA libraries were loaded onto the flow cell channels of an Illumina HiSeqTM 2000 platform for paired-end 90 bp × 2 sequencing at the Beijing Genomics Institute (BGI), Hongkong, China. Clean reads were obtained after removing reads containing adaptor sequences. The RNA reads have been deposited at NCBI under the bioproject No. PRJNA421992 with the SRA accession no. SRP126698.

### Reads mapping and differential expression analysis

The clean reads were firstly aligned to the *Xac* strain 306 genome (https://www.ncbi.nlm.nih.gov/nuccore/AE008923.1/) [24] using bowtie2 [25] with default parameters. The *in planta* differential expressed genes between wild type *Xac* 306 and ∆*rpfF* mutant strains were identified using DESeq2 R package [26] with the following cutoffs: |fold change| ≥ 2 and agjust-*P* ≤ 0.05. After aligned to *Xac* strain 306 genome, the remaining reads from each sample were analyzed mainly following the tuxedo pipeline [27]. Briefly, the reads were aligned to the sweet orange genome [28] using Tophat (v2.0.13) [29], and the generated alignments were fed to Cufflinks (v2.2.1) for transcript assembly [30]. The assemblies were combined with the sweet orange annotations using the cuffmerge algorithm and then fed to the cuffdiff2 for differentially expressed gene calling. Only the genes with |fold change| ≥ 2, q-value ≤0.05 and FPKM≥1 were considered as significantly differentially expressed genes (DEGs) between wild type strain infected and ∆rpf mutant strain infected plants. The MapMan gene functional categories were assigned to the DEGs using Mercator [30, 31] and the differentially regulated bins were identified by using MapMan [32].

### Functional annotation and classification

For citrus DEGs, the corresponding reference ID were obtained by blasting them to CitrusPLEX in plant expression database (PLEXdb, http://www.plexdb.org/plex.php?database=Citrus) [33]. Gene Ontology (GO) enrichment analysis of functional significance was applied to map all DEGs to terms in the agriGO (http://bioinfo.cau.edu.cn/agriGO/) database [34], looking for significantly enriched GO terms in DEGs. For bacterial DEGs, Clusters of Orthologous Groups (COG, https://www.ncbi.nlm.nih.gov/COG/) enrichment analysis was performed by comparing the prevalence of DEGs assigned to a specific COG category to the prevalence of genes in the whole genome assigned to that COG category with a Fisher’s exact test.

### Validation of RNA-seq results by qRT-PCR

To verify the RNA-seq result, qRT-PCR assays were conducted using the same set of RNA samples as for RNA-seq analysis. The aliquoted RNA sample (1 μg) used for RNA-seq was reverse transcribed using a QuantiTect Reverse Transcription kit with random hexamer primers (Qiagen, Valencia, CA) for two-step qRT-PCR. The gene specific primers (Additional file 1: Table S1) were designed to generate amplicons of 70 to 150 bp based on the DEGs sequences of citrus plant and *Xac* strain 306. qRT-PCR was conducted using QuantiTect SYBR Green PCR Kit (Qiagen, Valencia, CA) and the 7500 fast real-time PCR system (Applied Biosystems, Foster City, CA). Melting curve analysis of the PCR products was performed at the end of each PCR cycle to confirm the amplicon specificity. The housekeeping gene *CtGAPDH* [35] and *gyrA* [5] was used as plant and bacterial endogenous control, respectively. All reactions were repeated with three independent biological replicates and two technical replicates. The relative fold change in target gene expression was calculated by using the formula 2^-△△CT^ [36].

### Statistical analysis

Quantitative data were expressed as mean ± S.E.M. Statistical differences were evaluated through *t-test* and the level of statistically significant difference was set at *P*<0.05. All statistics were conducted using SAS 9.1.3.

## Results

### Canker progression and symptoms in inoculated citrus plants

**Fig. 1:**
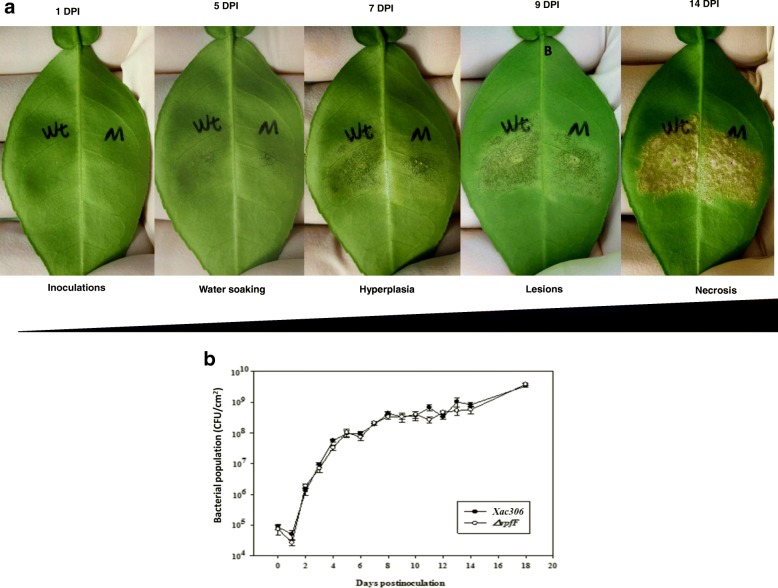
Citrus canker disease development in *Xanthomonas citri* inoculated Duncan grapefruit plants. **a** Representative leaves from ten replicates to show the development of canker symptoms on leaves inoculated with *Xanthomonas citri* subsp. *citri* wild type (Wt) strain 306 and its *∆rpfF *mutant (M) with bacterial solutions (5 × 10^6^ CFU/ml) by infiltration using needleless syringes and photographed at different days post inoculation (DPI). **b** Bacterial population growth in in Duncan grapefruit leaves inoculated with bacteria (5 × 10^6^ CFU/ml) at different days post inoculation. Error bars represent standard deviation. All the experiments were repeated three times

Duncan grapefruit (*Citrus paradisi* Macfadyen) seedlings were inoculated with *Xac* wild type strain 306 and its DSF deficient (∆*rpfF*) mutant strain for the development of typical symptoms of citrus canker. The first visible symptom, a water soaking area of the inoculated leaf, was observed at 5 days post inoculation (DPI). Within 14 DPI, typical symptoms of the canker disease were recorded (Fig. 1a): inoculated areas were characterized with water soaking, and then exhibited hyperplasia and hypertrophic, necrotic, erumpent lesions, as evidenced by the raised pustules. The ∆*rpfF* mutant produced weaker water soaking phenotypes compared to wild type strain 306 at 5 DPI under the tested conditions; and this becomes more evident at 7DPI and until 9 DPI (Fig.1a). However, the bacterial populations of the ∆*rpfF* mutant *in planta* were not significantly lower than the wild type strain (Fig. 1b).

### Sequencing the early citrus canker transcriptome

As the differences in the development of canker symptoms only were recorded in early stages of disease development (formation of water-soaking phenotypes) between the wild type Xac and ∆rpfF mutant (Fig. 1a), we speculated that the DSF/Rpf QS play certain role(s) in early stages of the Xac-citrus compatible interaction. Therefore, the early canker transcriptome during the formation of water-soaking phenotypes was profiled at 5 DPI.A total of 227 million and 278 million paired-end reads for wild type *Xac* strain 306 infected and the ∆*rpfF* mutant infected plants were produced respectively (Additional file 2: Table S2). All reads were aligned against the *Xac* strain 306 genome [24]. For each RNA-seq library, 2.4–5.5% of the reads mapped to the *Xac* 306 reference. Then the remaining *Xac* strain 306-unaligned reads were mapped against the sweet orange (*Citrus sinensis*) genome [28]*,* for the analysis of the citrus host transcriptome. A significant fraction of the *Xac*306-unaligned reads (63–68%) from both wild type *Xac* infected and ∆*rpfF* mutant infected libraries mapped to the sweet orange reference (Additional file 2: Table S2). Finally, of the 4374 *Xac* genes, 202 (4.5%) were determined as significantly differentially expressed genes (DEGs) [a minimum absolute value of a log2-fold change greater than 1 (equivalent to two-fold)] between wild type *Xac* 306 and ∆*rpfF* mutant strains in the conditions analyzed (Additional file 3: Table S3). Among them, 138 were upregulated in *Xac* wild type strain 306 compared to ∆*rpfF* mutant and 64 were downregulated. Of the 29,445 citrus (sweet orange) genes, 1946 genes were identified as significantly DEGs between wild type *Xac* 306 infected and ∆*rpfF* mutant strain infected plants, with 708 genes upregulated and 1238 downregulated in the wild type *Xac* 306 infected plants compared to ∆*rpfF* mutant strain infected plants (Additional file 4: Table S4).

**Fig. 2:**
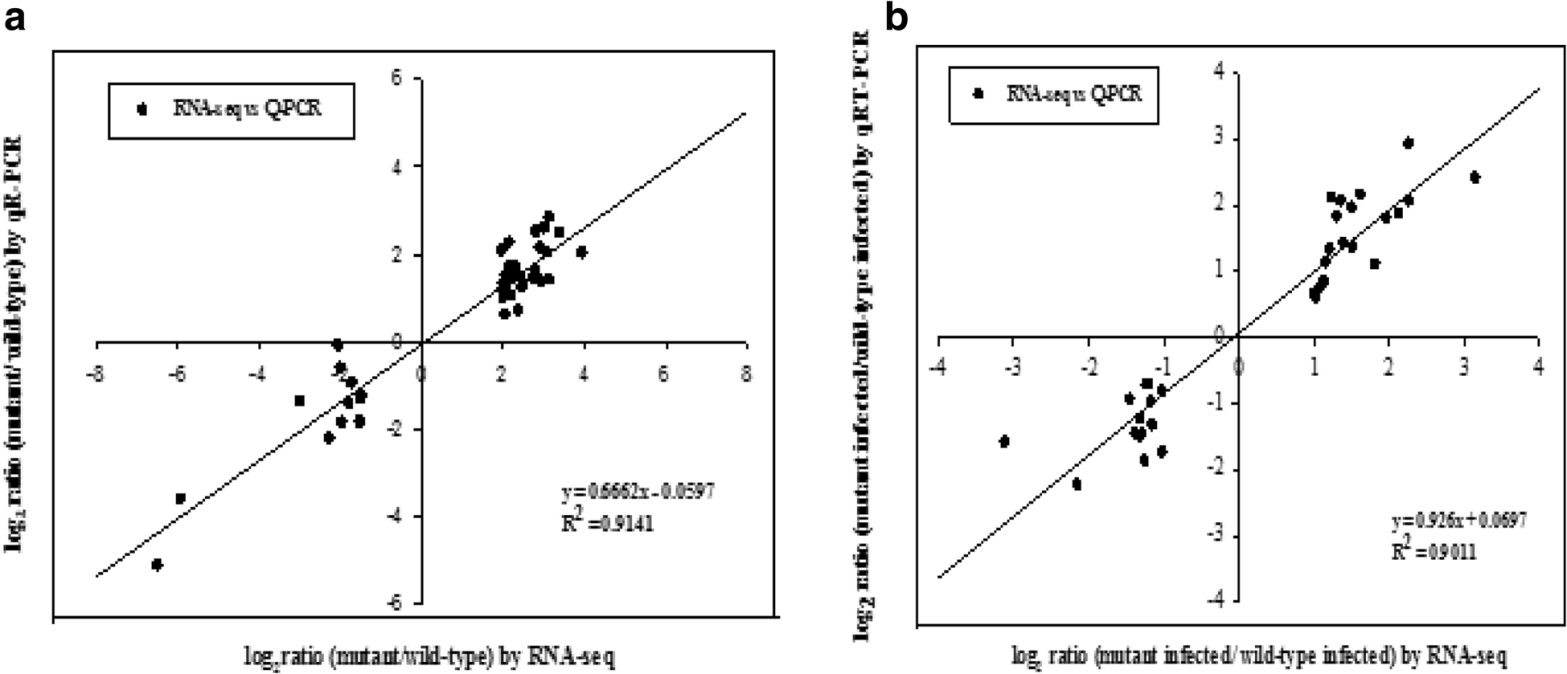
Correlation analysis of expression levels of selected bacterial genes and citrus genes determined by RNA-seq and RT-qPCR. **a** Comparison of RNA-seq and qRT-PCR data for differentially expressed genes (DEGs) in *Xanthomonas citri* subsp. *citri*. Fold changes were calculated for 40 bacterial genes and a high correlation (R^2^ > 0.90) was observed between the results obtained using the two techniques. **b** Comparison of RNA-seq and qRT-PCR data for DEGs in citrus. Fold changes were calculated for 33 citrus genes and a high correlation (R^2^ > 0.90) was observed between the results obtained using the two techniques

To validate the gene expression values obtained by RNA-seq, the expression of 40 *Xac* genes and 33 citrus genes (Additional file 1: Table S1) in the RNA samples used in RNA-seq analysis were analyzed by qRT-PCR assays. A strong correlation (R^2^ = 0.9141 for *Xac* gene expression; R^2^ = 0.9011 for citrus gene expression) were observed between the data produced by the two approaches (Fig. 2a-b), demonstrating the reliability of the results obtained.

### Functionally categorizing of *Xac* genes regulated by the DSF/Rpf-mediated QS system during early stages of host infection

**Fig. 3:**
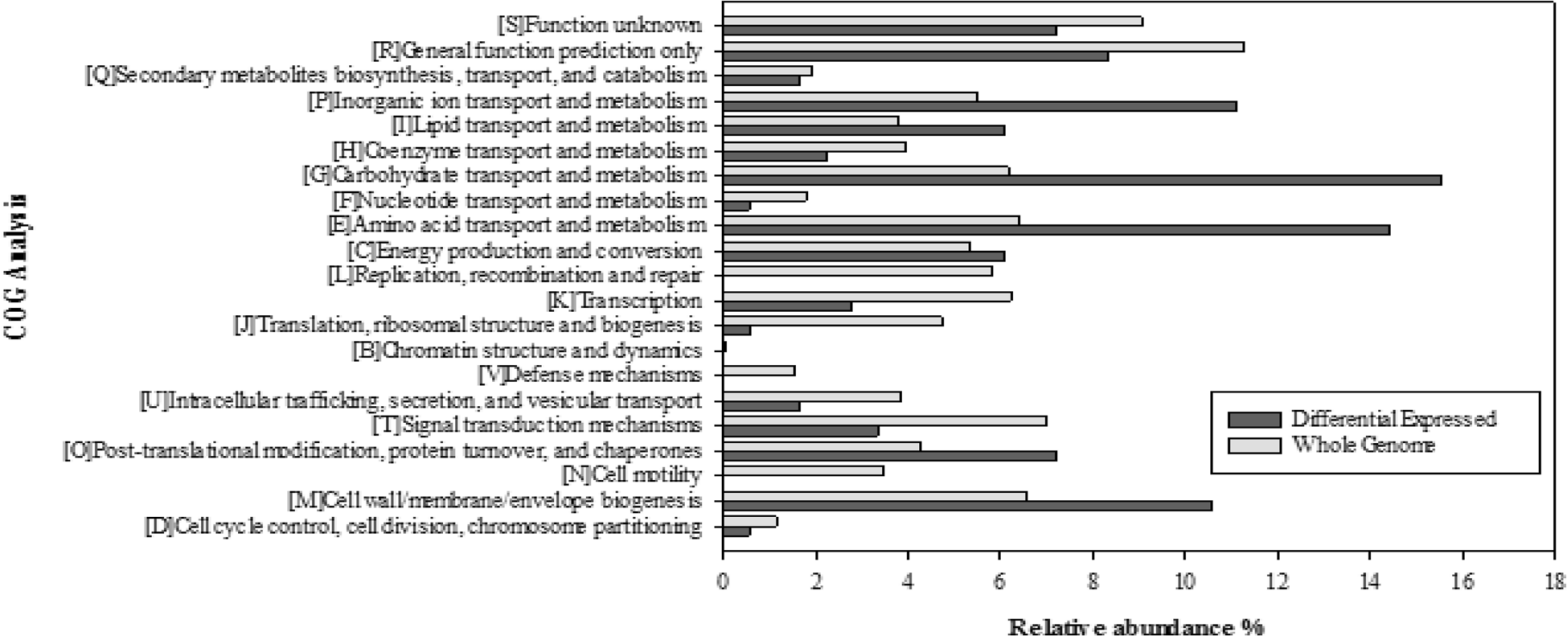
Distribution of differentially expressed genes (DEGs) of *Xanthomonas citri* subsp. *citri* in COG functional categories. The x-axis represents the relative abundance (%) of DEGs and all the genes in the bacterial whole genome in each COG category. The y-axis represents the functional classification each COG category

The 202 DEGs of *Xac* were subject to functionally categorizing with enrichment analyses of clusters of orthologous groups (COGs). The results showed that overrepresented COGs terms were mostly related to ‘Carbohydrate transport and metabolism’ (39 members, 16.3%), ‘Amino acid transport and metabolism’ (29 members, 14.4%), ‘Inorganic ion transport and metabolism’ (21 members, 10.4%), and ‘Cell wall/membrane/envelope biogenesis’ (19 members, 9.41%) (Fig. 3). Other enriched terms included ‘Lipid transport and metabolism’, ‘Energy production and conversion’, ‘Post-translational modification, protein turn over, and chaperones’, ‘signal transduction mechanisms’, ‘Transcription’, and ‘General function prediction only’. In addition, the genes annotated as hypothetical proteins were assigned to the ‘Function unknown’ group.

### DSF/Rpf-mediated QS regulates stress tolerance of *Xac* during early stages of host infection

A total 12 genes encoding enzymes involved in detoxification and stress tolerance of *Xac* at early stages of host infection were differentially regulated by DSF/Rpf**-**mediated QS (Table 1). Of these, the genes coding for a putative arabinose efflux permease belonging to the Major Facilitator Superfamily (MFS) transporter for sugar/drug (*araJ /*XAC1363), for a drug resistance translocase (*yieO /*XAC2494), for an endoproteinase (*argC*/XAC2992), and for trehalose biosynthesis (XAC0425 and XAC0429) were upregulated by ≥2-fold on average. Bacterial endoproteinases are able to degrade host defense proteins [37, 38], and trehalose protects bacterial cells from osmotic and oxidative stresses [39, 40]. The *katE* gene (XAC1211) encoding a catalase important for hydrogen peroxide torelance in Xac [41], was also upregulated by 2-fold.

**Table 1:** List of genes related to stress tolerance in *Xac* regulated by DSF/Rpf-mediated QS during early stages of host infection

Locus tag	Gene name	Log_2_Fold Change (Wt/∆*rpfF*)	Annotation/ Protein function
XAC0425	*glgA*	1.03	glycogen synthase (trehalose biosynthesis)
XAC0429	*glgY*	1.04	malto-oligosyltrehalose synthase
XAC1211	*katE*	1.00	catalase
XAC1363	*araJ*	1.32	arabinose efflux permease, MFS transporter
XAC1927	*aslB*	1.14	Fe-S oxidoreductase, stress-responsive
XAC2494	*yieO*	1.29	drug resistance translocase
XAC2992	*argC*	2.98	endoproteinase Arg-C, degrading host defense proteins
XAC4259	*blc*	1.05	lipocalin, involved in detoxification processes
XAC0906	*ahpF*	−1.01	alkyl hydroperoxide reductase scavenging H_2_O_2_
XAC0907	*ahpC*	−1.14	alkyl hydroperoxide reductase scavenging H_2_O_2_
XAC3486	*fabG*	−3.14	3-ketoacyl-ACP reductase, induced by nutrient limit conditions
XAC4361	*ttuB*	−1.51	MFS transporter

### DSF/Rpf -mediated QS is implicated in the regulation of nutrition utilization of *Xac* during early stages of host infection

A significant portion of the *Xac in planta* transcriptome regulated by DSF/Rpf -mediated QS is dedicated to nutrition utilization (Fig. 3). Of the 39 genes involved in carbohydrate uptake and metabolism, 14 were positively regulated by DSF/Rpf -mediated QS, while 25 were negatively regulated (Table 2). The carbohydrate genes upregulated by DSF/Rpf –mediated QS included those encoding cellulose endoglucanase (*egl*/XAC0029 and *engXCA/*XAC0612)*,* glycosyl transferase (*gtrB/*XAC1038/XAC2125*,* XAC3533, and *ugt/*XAC3921*),* glycosyl hydrolase (XAC3073), glycogen synthase (*glgA* /XAC0425 and *glgY*/XAC0429), glucose dehydrogenase (*gcd/*XAC1633/XAC3212*),* glucokinase (*glk*/XAC3120), and transporter (*araJ/*XAC1363 and *yieO/*XAC2494*).* In contrast, the expressions of *fruBK* and *fruA* encoding components of a fructose-specific phosphoenolpyruvate (PEP): carbohydrate phosphotransferase system (PTS) were downregulated.

**Table 2:** List of genes involved in nutrient transport or metabolism in *Xac* regulated by DSF/Rpf-mediated QS during pathogenic process

Locus tag	Gene name	Log_2_Fold Change (Wt/∆*rpfF*)	Annotation/ Protein function
Carbohydrates transport and metabolism			
XAC0029	*egl*	1.34	cellulase
XAC0425	*glgA*	1.03	glycogen synthase
XAC0429	*glgY*	1.04	malto-oligosyltrehalose synthase
XAC0612	*engXCA*	1.53	cellulase
XAC1038	*gtrB*	1.12	glycosyl transferase
XAC1363	*araJ*	1.32	MFS transporter
XAC1633	*gcd*	2.06	glucose dehydrogenase
XAC2125	*gtrB*	1.07	glycosyl transferase
XAC2494	*yieO*	1.29	drug resistance translocase
XAC3073		1.00	GH18 family; chitinase-like glycosyl hydrolase
XAC3120	*glk*	1.36	glucokinase
XAC3212	*gcd*	1.05	glucose dehydrogenase
XAC3533		1.23	Glycosyltransferase, GT2 family
XAC3921	*ugt*	1.52	glucosyltransferase
XAC0217	*lgtB*	−1.06	glycosyltransferase
XAC0299		−2.16	polysaccharide /chitin deacetylase
XAC0575	*ganB*	−1.98	arabinogalactan endo-1,4-beta-galactosidase
XAC1286	*abfA*	−1.09	alpha-L-arabinofuranosidase
XAC1308	*bga*	−1.18	beta-galactosidase
XAC1309	*galA*	−1.49	arabinogalactan endo-1,4-beta-galactosidase
XAC1556	*fucP*	−1.43	glucose-galactose transporter
XAC1557	*scrK*	−1.49	fructokinase
XAC1558		−1.46	putative N-acylglucosamine 2-epimerase
XAC1770	*celA*	−1.03	cellulase
XAC1771		−1.02	sialic acid-specific 9-O-acetylesterase
XAC1793	*celD*	−2.46	glucan 1,4-beta-glucosidase
XAC1794	*folk*	−2.38	sodium/glucose cotransport protein
XAC1812	*hmsF*	−1.72	HmsF protein /Polysaccharide deacetylase
XAC1813	*hmsH*	−2.06	HmsH protein /substrate-specific transmembrane transporter
XAC2501	*fruB*	−1.73	multiphosphoryl transfer protein
XAC2502	*fruK*	−1.68	1-phosphofructokinase
XAC2503	*fruA*	−1.79	PTS system fructose-specific transporter subunit II
XAC3474	*cit1*	−1.08	citrate carrier protein
XAC3487	*cebR*	−2.20	transcriptional regulator
XAC3489	*fyuA*	−1.49	TonB-dependent sucrose outer membrane transporter
XAC3490		−1.22	amylosucrase or alpha amylase
XAC4195	*ndvB/celAP*	−1.23	NdvB protein/ cellobionic acid phosphorylase
XAC4355		−1.34	Glyco_hydro like
XAC4361	*ttuB*	−1.51	MFS transporter
Amino acid transport and metabolism			
XAC0336	*metE*	1.72	5-methyltetrahydropteroyltriglutamate-methyltransferase
XAC0465		1.37	metalloproteinase
XAC1214	*gcvP*	1.09	glycine dehydrogenase
XAC2547	*dapA*	1.06	dihydrodipicolinate synthetase
XAC4326	*uahA*	6.50	urea amidolyase
XAC4327	*uahA*	5.92	allophanate hydrolase
XAC0174	*phhA*	−1.14	phenylalanine 4-monooxygenase
XAC0204	*glnA*	−3.39	glutamine synthetase
XAC0205	*glnB*	−3.01	nitrogen regulatory protein P-II
XAC0206	*amtB*	−2.78	ammonium transporter
XAC0300	*pucG*	−2.08	serine-pyruvate aminotransferase
XAC0301	*amaB*	−2.72	allantoate amidohydrolase
XAC1433	*asnB*	−1.19	asparagine synthetase B
XAC1820	*thrA*	−1.24	bifunctional aspartokinase I/homoserine dehydrogenase I
XAC1821	*thrB*	−1.20	homoserine kinase
XAC1823	*thrC*	−1.24	threonine synthase
XAC1828	*hisG*	−2.32	ATP phosphoribosyltransferase
XAC1829	*hisD*	−2.02	histidinol dehydrogenase
XAC1830	*hisC*	−1.94	histidinol-phosphate aminotransferase
XAC1831	*hisB*	−1.73	imidazole glycerol-phosphate dehydratase/phosphatase
XAC1832	*hisH*	−1.36	imidazole glycerol phosphate synthase subunit HisH
XAC1833	*hisA*	−1.61	1-(5-phosphoribosyl)-5- imidazole-4-carboxamide isomerase
XAC1834	*hisF*	−1.51	imidazole glycerol phosphate synthase subunit HisF
XAC1835	*hisI*	−1.12	phosphoribosyl-AMP cyclohydrolase
XAC3451	*ilvC*	−2.15	ketol-acid reductoisomerase
XAC3452	*ilvG*	−1.69	acetolactate synthase 2 catalytic subunit
XAC3453	*ilvM*	−1.49	acetolactate synthase isozyme II small subunit
XAC3454	*tdcB*	−1.71	threonine dehydratase
XAC3455	*leuA*	−1.22	2-isopropylmalate synthase
Lipid transport and metabolism			
XAC0159	*estA1*	1.15	carboxylesterase type B
XAC1037		1.12	membrane protein
XAC1316	*mmsB*	1.03	3-hydroxyisobutyrate dehydrogenase
XAC0375	*aes*	−1.42	lipase
XAC2012	*fadA*	−1.25	acetyl-CoA acetyltransferase
XAC2013	*fadB*	−1.66	3-hydroxyacyl-CoA dehydrogenase
XAC3300	*estA*	−1.10	esterase
XAC3486	*fabG*	−3.14	3-ketoacyl-ACP reductase
XAC3959		−1.69	Acyl-CoA delta-9-desaturase
Inorganic ion transport and metabolism			
XAC1578	*phoX*	1.34	phosphate-binding protein
XAC1579	*oprO*	1.50	polyphosphate-selective porin O
XAC0296		−2.50	monoxygenase
XAC0310	*vanB*	−3.94	vanillate O-demethylase oxidoreductase
XAC0311	*vanA*	−3.07	vanillate O-demethylase oxygenase
XAC0742		−1.45	RcnB containg protein
XAC0999	*cirA*	−1.04	colicin I receptor
XAC3168	*bfeA*	−1.55	ferric enterobactin receptor
XAC3169	*bfeA*	−1.17	ferric enterobactin receptor
XAC3472	*oprO*	−1.82	polyphosphate-selective porin O
XAC3484	*oprO*	−2.90	porin

For the genes involved in uptake and metabolism of amino acids, most (23 out of 29) were downregulated by DSF/Rpf QS *in planta*, while a small portion (6/29) were upregulated (Table 2). Among the downregulated genes, some are involved in the biosynthesis of asparagine (*asnB/*XAC1433), tyrosine (*phhA/*XAC0174), glutamine (*glnA/*XAC0204 and *glnB/*XAC0205), glycine (*pucG/*XAC0300 and *amaB/*XAC0301), threonine (*thrAB/*XAC1820, XAC1821*,* and *thrC/*XAC1823)*,* histidine (*hisGDCBHAFI/*XAC1828–1835*),* and biosynthesis of isoleucine, leucine, and valine (*ilvCGM, tdcB, leuA/*XAC3451–3455)*.* Over-presented in the up-regulated genes are those for biosynthesis of methionine (*metE/XAC0306*) and lysine (*dapA/*XAC2547)*,* for a metalloproteinase (XAC0465), and for glycine biosynthesis and cleavage (*gcvP/*XAC1214*).* Remarkably, the urea amidolyase and an allophanate hydrolase, which catalyze the release of ammonia from urea, showed distinctive expression levels (Log_2_Fold Change ≥5.9) upregulated by DSF/RPF QS in *Xac* during host infection.

Eleven differentially expressed genes were related to inorganic ion transport and metabolism in *Xac* during host infection (Table 2). Remarkably, the two genes (*phoX*/ XAC1578 and *oprO*/XAC1579) encoding phosphate transporter proteins were upregulated by an average of 2.6-fold by DSF/Rpf QS during infection. The genes for siderophore biosynthesis (*entF/*XAC3922) and for iron storage protein in the bacterioferritin family (*bfr/*XAC1149) [42] were upregulated two-fold or more (Table 3). Six genes encoding TonB-dependent outer-membrane receptors involved in siderophore-mediated ferric iron uptake by *Xac* [42, 43], including *fecA/*XAC0690*, btuB/*XAC1310, and *fyuA/*XAC3489, were downregulated two-fold on average. In addition, the two genes coding for ferric enterobactin receptors involved in siderophore uptake (*bfeA*/ XAC316*8* and XAC 3169) were also downregulated two-fold on average (Table 3).

**Table 3:** List of ferric iron uptake genes in *Xac* regulated by DSF/RPF during pathogenic process

Locus tag	Gene name	Log_2_Fold Change (Wt/∆*rpfF*)	Annotation/ Protein function
XAC1149	*bfr*	1.01	Bacterioferritin, iron storage
XAC3922	*entF*	1.42	ATP-dependent serine activating enzyme (nonribosomal peptide synthetases, siderophore biosyntensis)
XAC0690	*fecA*	−1.08	TonB-dependent outer membrane receptor
XAC1310	*btuB*	−2.07	TonB-dependent outer membrane receptor
XAC1768	*fhuA*	−1.19	TonB-dependent outer membrane receptor
XAC1769	*cirA*	−1.71	TonB-dependent outer membrane receptor
XAC2312		−1.27	TonB-dependent outer membrane receptor
XAC3489	*fyuA*	−1.49	TonB-dependent outer membrane receptor
XAC3168	*bfeA*	−1.55	Ferric enterobactin receptor, siderophore
XAC3169	*bfeA*	−1.18	Ferric enterobactin receptor, siderophore

### Genes for signal transducers and/or transcriptional regulators regulated by DSF/Rpf-mediated QS in *Xac* during early stages of host infection

The expression of 12 genes coding for signal transducers and/or transcriptional regulators in *Xac* were differentially regulated by DSF/Rpf-mediated QS (Table 4). Of these, two genes were upregulated and 10 genes were downregulated. The two upregulated genes were *XAC1328* and *XAC3927,* encoding a putative CheY-like superfamily protein and serine/threonine protein kinase respectively, both are of signal transducer activity. Among those genes downregulated were the two genes *ntrB* (XAC0207) and *ntrC* (XAC0208) encoding the NtrB/C two-component system, which interacts with the RpfC/G system responding to DSF signal to regulate *sigma54*-dependent promoters in vitro [44]. In addition, the two-component sensor genes *tctE* (XAC3482) and XAC3720, the transcriptional regulator genes *acoR* (XAC0654), *tetR* (XAC2014), *iscR* (XAC 2934), and *cebR* (XAC3487), the AbrB ambiactive repressor and activator (XAC1883), and the Trp operon repressor gene (*trpR*/XAC1827) were downregulated by the DSF/Rpf –mediated QS. The homologues of these signal transduction and transcription factors constitute regulators of virulence and adaptation factors in many bacteria, including the human bacterial pathogens enterotoxigenic *E. coli* [45] and *P. aeruginosa* [46], and the model organism *Bacillus subtilis* strain 168 [47]. For example, the IscR transcriptional repressor in *E. coli* negatively controls the type I fimbriae colonization factor synthesis and biofilm formation in response to both iron limitation and oxidative stress [45]. The *trp* repressor negatively regulates expression of genes involved in tryptophan biosynthesis, transport, and metabolism in response to intracellular levels of tryptophan, but also regulates transcription initiation in several other operons related to tryptophan metabolism that are important for expression of virulence factors in *E. coli* and *P. aeruginosa* [46]. Thus, they might function as regulators of virulence and adaptation factors in *Xac* by modulating biofilm formation and adhesion factor production, which are crucial for attachment and colonization of the tissues and for consequent invasion [17].

**Table 4:** Summary of *Xac* DEGs coding for signal transduction and transcriptional factors regulated by DSF/Rpf-mediated QS during pathogenic process

Locus tag	Gene name	Log_2_Fold Change (Wt/∆*rpfF*)	Annotation/ Protein function
XAC1328		1.07	CheY-like protein superfamily
XAC3927		1.04	serine/threonine protein kinase
XAC0207	*ntrB*	−1.28	two-component system sensor protein
XAC0208	*ntrC*	−1.21	two-component system regulatory protein
XAC0654	*acoR*	−1.27	transcriptional regulator AcoR
XAC1827		−2.41	hypothetical protein/ Trp repressor protein (represses transcription of the Trp operon)
XAC1883		−1.00	hypothetical protein/ AbrB domain containing transcriptional regulator
XAC2014		−1.29	TetR family transcriptional regulator
XAC2934	*iscR*	−1.02	hypothetical protein/ Iron-sulfur cluster regulator IscR (Fe-S assembly SUF system transcriptional regulator)
XAC3482	*tctE*	−1.02	two-component system sensor protein
XAC3487	*cebR*	−2.20	transcriptional regulator
XAC3720		−1.18	hypothetical protein/ putative two-component system sensor kinase

### Putative function of the hypothetical protein encoding genes within the DSF/Rpf-mediated QS regulon *in planta*

BLASTx analysis showed that 39 of the 44 genes within the DSF/Rpf QS regulon encoding hypothetical proteins had significant similarities only to sequences in bacteria within the *Xanthomonas* genus. Based on sequence similarity and conserved domain detected, we defined putative functions for 24 of the 44 genes, which are potentially involved in bacterial adaptation and pathogenesis (Table 5). Some of these genes encode proteins with recognized roles in bacterial pathogenesis, such as members of the cell surface adhesion protein families (XAC3546) and chemotaxis protein families (XAC3753 and XAC3754). Interestingly, the genes encoding stress-induced protein (XAC2156) and Ferritin-like di-iron-carboxylate protein (XAC2155) were upregulated by DSF/Rpf –mediated QS and possibly involved in the adaptation of *Xac* to the host environment. The genes encoding putative GH18_chitinase-like glycosyl hydrolase (XAC3073) and GT2 family glycosyltransferase (XAC3533) were also upregulated, involved in carbohydrate transport and metabolism. In contrast, the gene XAC3085 encoding a putative T3SS effector protein was downregulated, with an unknown function in *Xac*-citrus interaction.

**Table 5:** Summary of *Xac* DEGs encoding hypothetical proteins regulated by DSF/Rpf-mediated QS during pathogenic process

Locus tag	Log_2_Fold Change (Wt/∆*rpfF*)	Homologue [Bacterial species]	Identity (%)^c^
XAC2155	1.36	ferritin-like domain-containing protein [*Xanthomonas* group]	99
XAC2156	1.97	stress-induced protein [*X. phaseoli*]	98
XAC3073	1.01	GH18_chitinase-like glycosyl hydrolase [*X. citri*]	99
XAC3533	1.23	glycosyltransferase, GT2 family [*X. axonopodis*]	97
XAC3546	1.29	autotransporter adhesion protein [*X.citri*]	99
XAC0295	−1.64	5-hydroxyisourate hydrolase [*X. citri*]	98
XAC0297	−2.93	2-oxo-4-hydroxy-4-carboxy-5-ureidoimidazoline decarboxylase [*X. citri*]	99
XAC0298	−1.84	Nuclear transport factor 2 (NTF2-like) superfamily [*X. axonopodis*]	99
XAC0510	−1.22	FUSC-like inner membrane protein (fusaric acid resistance) [*X. citri*]	98
XAC1397	−2.05	Alginate export domain containing protein [*X. axonopodis*]	99
XAC1471	−1.12	Glycine zipper 2TM domain containing protein [*X. citri*]	98
XAC1827	−2.41	Trp repressor protein [*Xanthomonas* group]	99
XAC1883	−1.00	AbrB domain containing transcriptional regulator [*X. citri*]	99
XAC1884	−1.26	PIN (PilT N terminus) domain-containing protein [*X. citri*]	99
XAC2821	−1.02	Crotonase/Enoyl-Coenzyme A (CoA) hydratase [*Xanthomona*s group]	99
XAC2934	−1.02	Fe-S assembly SUF system transcriptional regulator [*X. citri*]	99
XAC3085	−1.06	putative type III secretion system effector protein [*Xanthomonas* group]	99
XAC3439	−1.16	putative secreted protein [*Xanthomonas* group]	99
XAC3506	−1.67	Cellulose belonging to glycosyl hydrolase family 5 [*X. citri*]	98
XAC3507	−1.99	CelS cellulose; Glycosyl hydrolase 12 superfamily [*Xanthomonas* group]	98
XAC3720	−1.17	putative two-component system sensor kinase [*Xanthomonas* group]	99
XAC3753	−1.22	putative chemotaxis membrane protein [*Xanthomonas* group]	99
XAC3754	−1.01	putative chemotaxis membrane protein [*Xanthomonas* group]	99
XAC3856	−1.19	calcium-binding protein, EFh Superfamily [*X. citri*]	99
XAC4219	−1.09	Lipid-binding SYLF domain containing protein [*Xanthomonas* group]	99

### Comparison of the DSF/Rpf-mediated QS regulons *in planta* and in vitro

Our previous work identified 180 genes constituting the DSF/RpfF regulon of *Xac* grown in culture medium in the exponential and/or stationary growth phase [17]. Among those, a set of 31 genes were overlapping with the *in planta* DSF/Rpf regulon, 26 of which showed similar trends in alteration of expression between the two environmental conditions (Additional file 5: Table S5). Specifically, a subset of 20 genes were identified in the DSF/RpfF regulon of *Xac* in the exponential growth phase, 25 genes were identified in the DSF/RpfF regulon of *Xac* in the stationary growth phase, and 14 genes were identified in both regulons. These genes were primarily involved in energy metabolism (carbohydrate transport and metabolism), protein fate and protein synthesis (amino acid transport and metabolism or post-translational modification), and signal transduction or transcriptional regulation, and some encode hypothetical proteins with unknown functions.

### Overview of citrus transcriptional responses to DSF/Rpf-mediated *Xac* infection

**Fig. 4:**
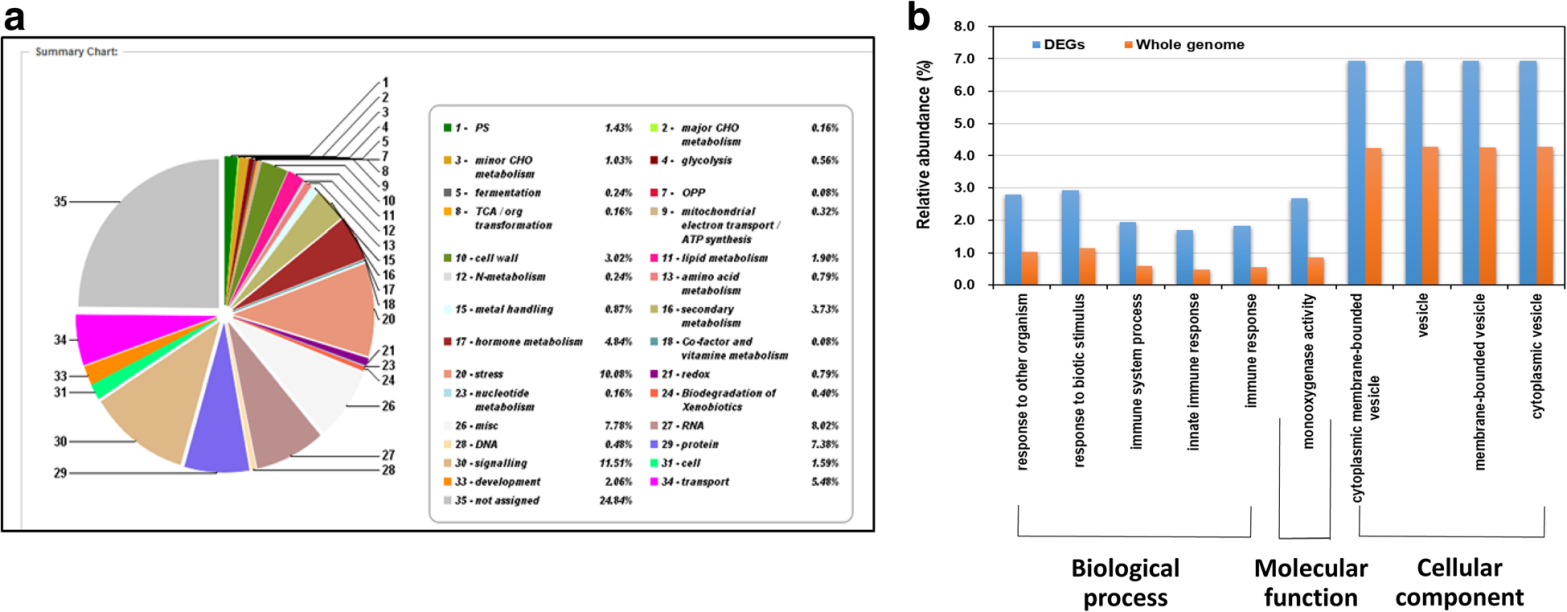
Gene Ontology classification of citrus differentially expressed genes (DEGs) response to the DSF/Rpf-mediated *Xanthomonas citri* subsp. *citri* infection. **a** Pie diagram depicting the relative abundance of each category of DEGs. The category was presented by functional classification followed by the corresponding percentage. **b** Column chart showing the relative abundance of the three main categories of DEGs: biological process, cellular component, and molecular function

**Fig. 5:**
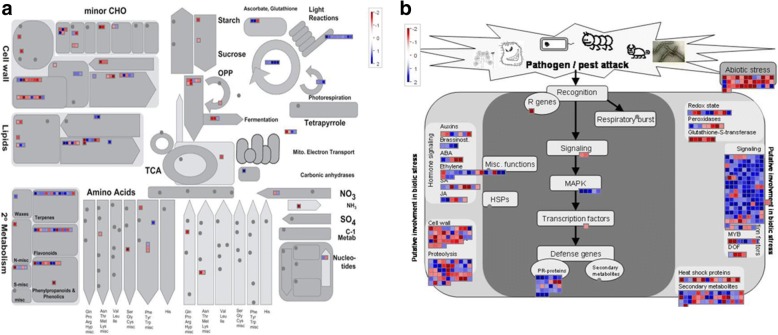
Display of citrus differentially expressed genes (DEGs) response to the DSF/Rpf-mediated *Xanthomonas citri* subsp. *citri* infection that are involved in different metabolic pathways (**a**) or biotic/abiotic stress responses (**b**). The log_2_ fold change of gene expression (∆*rpfF* -inoculated plants versus wild type *Xanthomonas citri* subsp. *citri* -inoculated plants) was analyzed using MapMan. Each square represents an individual gene within a category. Small squares colored in red and blue represent genes in infected plants that were up- and down-regulated by DSF/Rpf – mediated Xac infection, respectively. A false color scale was used and all the values were given on a log_2_ scale. The color saturates at a 4-fold change (i.e. log_2_ ratio = 2 or − 2). A significant downregulation was observed for many genes that are involved in photosynthesis, secondary metabolism, or response to biotic stress including genes for signaling, hormone metabolisms, and plant defense responses

Global analyses of the citrus transcripts in response to DSF/Rpf-mediated *Xac* infection revealed that the protein families related to stress responses, signaling pathways, hormone metabolism, and cell wall modification were over-represented according to the gene ontology (GO) analysis (Fig. 4). Individual gene responses in metabolic pathways were visualized using the MapMan tool (Fig. 5). Remarkable downregulation was observed for many genes related to photosynthesis, secondary metabolism, and plant defense response.

### DSF/Rpf -mediated *Xac* infection represses photosynthesis in citrus

The expression levels of nine genes involved in photosynthesis decreased significantly in wild type *Xac* strain 306 infected leaf tissues, compared with the ∆*rpfF* mutant infected leaf tissues (Table 6). Transcripts for photosystem II oxygen-evolving enhancer protein PsbO (Cs7g03508) and photosystem II 22 kDa protein PsbS (Cs3g19650) were less abundant in wild type *Xac* infected leaves. Three transcripts encoding subunits of photosystem I also decreased in wild type *Xac* infected leaves, including photosystem I reaction center subunit II (PsaD), VI-2 (PsaH), and O subunit (PsaO). In addition, the genes for photosynthetic electron transport protein plastocyanin (PetE) and for an ATP synthase subunit (the F-type H + −transporting ATPase subunit gamma, Cs4g10260) were downregulated in wild type *Xac* infected leaves (Table 6). These results are in agreement with the notion that *Xac* is biotrophic during early stages of host infection [48, 49] and that biotrophic pathogen infection generally represses photosynthesis in host plants [50].

**Table 6:** Summary of citrus DEGs genes involved in photosynthesis

ID	Gene name (Locus)	Log_2_Fold Change (Wt/∆*rpfF*)	Annotation/ Protein function
XLOC_017330	*psbQ* (Cs7g03580)	−1.36	photosystem II oxygen-evolving enhancer protein
XLOC_008489	*psbS* (Cs3g19650)	−2.10	photosystem II 22 kDa protein
XLOC_014472	*psaD* (Cs5g31180)	−1.18	photosystem I reaction center subunit II
XLOC_002098	*psaH* (Cs1g15170*)*	−1.14	photosystem I reaction center subunit VI-2
XLOC_015536	*psaO* (Cs6g12390)	−1.13	photosystem I subunit O
XLOC_008847	*petE* (Cs3g26730)	−1.22	photosynthetic electron transport protein plastocyanin
XLOC_004226	Cs2g26640	−1.27	GLK2 transcription factor, regulating the expression of photosynthetic apparatus
XLOC_010577	*atpA* (Cs4g10260)	−1.07	F-type H + −transporting ATPase subunit gamma
XLOC_001762	*psaN* (Cs1g09130)	1.23	photosystem I reaction center subunit N

### Alterations of hormone metabolisms in citrus responding to DSF/Rpf-mediated *Xac* infection

Significant transcriptional changes in response to DSF/Rpf-mediated *Xac* infection were observed for a group of genes related to plant hormone biosynthesis, transportation, metabolism, and associated signal transduction (Table 7). Transcripts for auxin biosynthesis-related enzymes and auxin-responsive proteins, including indole-3-acetate beta-D-glucosyltransferase (IAGLU), UDP-glucosyltransferase (UGT74E2), and SAUR (small auxin-up RNA) -like auxin-responsive protein, were more abundant, while genes for the PIN or PIN-LIKES class of auxin transporters were downregulated in wild type *Xac* infected leaves. The gene *Cs2g03270* encoding a 9-*cis*-epoxycarotenoid dioxygenase, a key enzyme for abscisic acid (ABA) biosynthesis [51], was downregulated in wild type *Xac* infected leaves, and gens for ABA-responsive (ABR) proteins were upregulated in wild type Xac infected leaves. Three genes involved in cytokinin biosynthesis (cytokinin synthase, isopentenyltransferase (IPT), and UDP-Glycosyltransferase superfamily protein) were downregulated in wild type *Xac* infected leaves, while two genes involved in cytokinin metabolic process (UDP-glucosyl transferase 85A5 (UGT85A5) and DON-Glucosyltransferase) were upregulated in wild type *Xac* infected leaves.

**Table 7:** Summary of citrus DEGs genes involved in plant hormone metabolisms

ID	Locus	Log_2_Fold Change (Wt/∆*rpfF*)	Annotation/ Protein function
Auxin biosynthesis, metabolism, and signaling			
XLOC_012174	Cs5g20410	1.22	Indole-3-acetate beta-D-glucosyltransferase (IAGLU)
XLOC_031022	–	1.38	Indole-3-acetate beta-D-glucosyltransferase (IAGLU)
XLOC_005577	Cs2g23750	2.45	UDP-glucosyltransferase acting on IBA (indole-3-butyric acid), affects auxin homeostasis
XLOC_029081	orange1.1 t02620	1.65	SAUR-like auxin-responsive protein family
XLOC_003150	Cs2g06290	1.00	Aluminium induced protein with YGL and LRDR motifs, auxin-responsive
XLOC_015754	Cs6g17000	−1.61	Probable auxin efflux carrier component 1c (PIN1c)
XLOC_020295	Cs7g31320	−1.19	Auxin transporter-like protein 1 (PIN-like protein 1)
XLOC_008042	Cs3g10670	−1.28	NAD(P)-linked oxidoreductase superfamily protein, auxin regulated
Abscisic acid (ABA) -related genes			
XLOC_004564	Cs2g03270	−1.21	9-cis-epoxycarotenoid dioxygenase for ABA biosynthesis
XLOC_004925	Cs2g10990	−1.71	UDP glycosyltransferase (UGT) for ABA biosynthesis
XLOC_017286	Cs7g02850	2.07	GRAM domain-containing protein, ABA-responsive protein-related
XLOC_017832	Cs7g13470	2.64	GRAM domain-containing protein, ABA-responsive protein-related
XLOC_012807	Cs5g32930	1.29	membrane-bound protein (*Arabidopsis thaliana* TSPO-related), induced by ABA
Ethylene - related genes			
XLOC_010327	Cs4g05190	1.48	flavanone 3 hydroxylase, 2-oxoglutarate (2OG) and Fe(II)-dependent oxygenase superfamily protein, involved in ethylene synthesis
XLOC_004668	Cs2g05280	1.08	ERF (ethylene response factor) subfamily B-3 of ERF/AP2 transcription factor family (ERF1)
XLOC_014405	Cs5g29870	1.86	ERF (ethylene response factor) subfamily B-3 of ERF/AP2 transcription factor family (ERF1)
XLOC_024633	–	1.36	ERF (ethylene response factor) subfamily B-3 of ERF/AP2 transcription factor family(ERF1)
XLOC_007284	Cs3g23270	1.79	DREB subfamily A-5 of ERF/AP2 transcription factor family (RAP2.1)
XLOC_005573	Cs2g23660	− 1.31	Ethylene-responsive transcription factor 4 (Ethylene-responsive element-binding factor 4 homolog) (EREBP-3)
XLOC_003119	Cs2g05620	−1.32	Ethylene-responsive transcription factor 4 (Ethylene-responsive element-binding factor 4 homolog) (EREBP-3)
XLOC_001696	Cs1g07950	−1.17	ERF (ethylene response factor) subfamily B-1 of ERF/AP2 transcription factor family (ERF-4)
XLOC_001450	Cs1g03280	−1.07	ERF (ethylene response factor) subfamily B-3 of ERF/AP2 transcription factor family (ERF13)
XLOC_014725	–	−2.28	ERF (ethylene response factor) subfamily B-3 of ERF/AP2 transcription factor family (ERF-6)
XLOC_024283	Cs9g13620	−2.24	ERF (ethylene response factor) subfamily B-3 of ERF/AP2 transcription factor family (ERF104)
XLOC_023353	Cs9g13610	−2.04	ERF (ethylene response factor) subfamily B-3 of ERF/AP2 transcription factor family (ERF104)
XLOC_003353	Cs2g09980	−1.39	Ethylene-responsive nuclear protein / ethylene-regulated nuclear protein (ERT2)
XLOC_028605	orange1.1 t01663	−1.38	Adenine nucleotide alpha hydrolases-like superfamily protein, involved in response to stress
XLOC_002875	Cs2g01100	−1.97	DUF247 domain containing plant protein, probably involved in ethylene signal transduction
XLOC_004471	Cs2g01150	−1.43	DUF247 domain containing plant protein, probably involved in ethylene signal transduction
XLOC_004467	Cs2g01090	−1.01	DUF247 domain containing plant protein, probably involved in ethylene signal transduction
XLOC_014014	Cs5g22160	−1.18	DUF247 domain containing plant protein, probably involved in ethylene signal transduction
Cytokinin - related genes			
XLOC_023917	Cs9g06010	−1.51	cytokinin synthase for cytokinin biosynthesis
XLOC_003491	Cs2g12620	−1.14	putative adenylate isopentenyltransferase (IPT), involved in cytokinin biosynthesis
XLOC_008154	Cs3g12960	− 1.59	UDP-Glycosyltransferase superfamily protein, involved in cytokinin biosynthesis
XLOC_030591	orange1.1 t05518	1.01	UDP-glucosyl transferase 85A5 (UGT85A5), involved in cytokinin metabolic process
XLOC_030963	–	1.47	DON-Glucosyltransferase, UDP-Glucosyl transferase superfamily protein, involved in cytokinin metabolic process
Gibberellic acid (GA)- related genes			
XLOC_019477	Cs7g14940	−1.17	gibberellin 2-oxidase (GA2OX), involved in gibberellin metabolic process
XLOC_028715	orange1.1 t01909	−1.81	CYP701A cytochrome p450 family protein, involved in gibberellin biosynthesis
XLOC_005279	Cs2g17800	−1.57	ARM (Armadillo-type fold) repeat superfamily protein, involved in GA signal transduction
XLOC_005280	Cs2g17820	−1.14	ARM (Armadillo-type fold) repeat superfamily protein, involved in GAsignal transduction
XLOC_008817	Cs3g26100	−1.11	GA-responsive GAST like protein
XLOC_006493	Cs3g07395	1.16	Gibberellin-regulated family protein
Salicylic acid (SA) - related genes			
XLOC_001130	Cs1g23160	1.00	Methyl salicylate (MeSA) esterase-like protein, involved in MeSA hydrolysis to SA
XLOC_005805	Cs2g28310	−1.04	S-adenosyl-L-methionine-dependent methyltransferases superfamily protein, involved in SA metabolic process
XLOC_016863	Cs6g18050	−1.33	S-adenosyl-L-methionine-dependent methyltransferases superfamily protein, involved in SA metabolic process
Jasmonic acid (JA) - related genes			
XLOC_029628	orange1.1 t03726	1.35	12-oxophytodienoic acid reductases, involved in JA biosynthesis
XLOC_029630	orange1.1 t03729	1.64	FMN-containing oxidoreductases, involved in JA biosynthesis
XLOC_020298	Cs7g31430	1.05	S-adenosyl-L-methionine: jasmonic acid carboxyl methyltransferase (JMT), involved in JA metabolic process to form methyljasmonate (MeJA)
XLOC_026677	orange1.1 t03773	−1.51	Chloroplast lipoxygenase required for wound-induced JA accumulation in Arabidopsis
XLOC_029950	orange1.1 t04376	−2.04	Chloroplast lipoxygenase required for wound-induced JA accumulation in Arabidopsis
XLOC_002571	Cs1g24440	−1.22	S-adenosyl-L-methionine: jasmonic acid carboxyl methyltransferase (JMT), involved in JA metabolic process to form methyljasmonate (MeJA)
Brassinosteroid (BR) - related genes			
XLOC_010301	Cs4g04730	−1.10	cycloartenol synthase 1 (CAS1), involved in the biosynthesis of BRs
XLOC_012247	Cs5g21830	−1.12	C-8 sterol isomerase, involved in the biosynthesis of BRs
XLOC_002765	–	− 2.43	Leucine-rich receptor-like protein kinase family protein, involved in BR signaling pathways
XLOC_006131	–	−1.52	Leucine-rich receptor-like protein kinase family protein, involved in BR signaling pathways

A total of 17 genes encoding the ethylene response factor (ERF) transcription factors were differentially expressed in wild type *Xac* infected leaves compared to ∆*rpfF* mutant infected leaves (Table 7). In particular, the transcripts for ERF1 and RAP2.1 were more abundant in wild type *Xac* infected leaves, while transcripts for EREBP-3, ERF-4, ERF-6, ERF104, and for an ethylene-regulated nuclear protein (ERT2) were less abundant in wild type *Xac* infected leaves. One gene (Cs4g05190) involved in ethylene biosynthesis was upregulated in wild type *Xac* infected leaves. Two genes for gibberellic acid (GA) biosynthesis (the CYP701A cytochrome p450 family protein) and GA inactivation (GA2OX: gibberellin 2-oxidase) [52] were downregulated in wild type *Xac* infected leaves (Table 7). Three genes involved in the GA response were also downregulated in wild type *Xac* infected leaves, including those GAST-like (gibberellic acid stimulated transcript-like) and ARM repeat superfamily proteins.

Three genes involved in jasmonic acid (JA) biosynthesis or metabolisms were upregulated in wild type *Xac* infected leaves compared to ∆*rpfF* mutant infected leaves (Table 7). These included the gene encoding 12-oxophytodienoic acid reductases (OPR) (orange1.1 t03726) and the gene encoding a FMN-containing oxidoreductases (orange1.1 t03729) (for JA biosynthesis), and a S-adenosyl-L-methionine:jasmonic acid carboxyl methyltransferase (JMT) that catalyzes the formation of methyl jasmonate (MeJA) from JA (Cs7g31430) [53]. In contrast, two genes (orange1.1 t03773 and orange1.1 t04376) encoding the chloroplast lipoxygenases required for wound-induced JA accumulation in *Arabidopsis* were downregulated in wild type *Xac* infected leaves. Three genes involved in SA metabolisms were differentially expressed in wild type *Xac* infected leaves compared to ∆*rpfF* mutant infected leaves (Table 7). Notably, a gene (Cs1g23160) encoding the methyl esterase 1 (MES1) with methyl salicylate (MeSA) esterase activity of hydrolyzing MeSA to SA *in planta* [54], was upregulated in wild type *Xac* infected leaves. In addition, two genes (Cs2g28310 and Cs6g18050) encoding S-adenosyl-L-methionine-dependent methyl transferases superfamily proteins involved in SA metabolic process were downregulated in wild type *Xac* infected leaves. Furthermore, four genes involved in brassinosteroid (BR) biosynthesis or responses were repressed in wild type Xac infected leaves. They are a cycloartenol synthase 1 (CAS1) and a C-8 sterol isomerase involved in the biosynthesis of BR, and two leucine-rich receptor-like protein kinase family proteins involved in BR signaling pathways [55] (Table 7).

### Citrus defense responses to DSF/Rpf-mediated *Xac* infection

Of the 1946 citrus DEGs between wild type *Xac* infected - and ∆*rpfF* mutant infected - libraries, 102 genes (5.4%) were identified to be involved in plant defense responses, with 32 genes upregulated and 70 genes downregulated by DSF/Rpf-mediated *Xac* infection (Additional file 6: Table S6; Table 8). Remarkably, 34 genes encoding plant immune receptor-like proteins or receptor-like kinases were downregulated. Eight genes encoding transcription regulators were downregulated, including three WRKY transcription factors- encoding genes (one for WRKY 4 and two for WRKY 53). In addition, four genes encoding pathogenesis-related (PR) family proteins were downregulated, including the genes encoding members of the PR-5 (thaumatin) and PR-6 (protease inhibitor) subfamily (Additional file 6: Table S6). Three Kunitz protease inhibitors encoding genes were also downregulated, which were suggested to modulate programmed cell death in in *Arabidopsis* during plant–pathogen interactions [56]. Other downregulated genes included five genes encoding NB-ARC (nucleotide-binding adaptor shared by Apaf-1, resistance proteins, and CED-4) domain-containing disease resistance proteins [57], and three genes encoding MYB transcription factor family proteins, which are involved in various plant biological processes including defense responses [58].

**Table 8:** Summary of citrus DEGs genes encoding putative immune receptors and transcription factors involved in plant defense responses

ID	Locus	Log_2_Fold Chang) (Wt/∆*rpfF*)	Annotation/ Protein function
Receptor encoding genes			
XLOC_030903	–	− 1.12	Receptor like protein 1 (RLP1), Leucine-rich repeat-containing
XLOC_028463	orange1.1 t01371	−1.36	Receptor like protein 1 (RLP1), Leucine-rich repeat-containing
XLOC_022248	Cs8g14810	−1.55	Receptor like protein 1 (RLP1), Leucine-rich repeat-containing
XLOC_007802	Cs3g06050	−1.99	Receptor like protein 1 (RLP1), Leucine-rich repeat-containing
XLOC_023272	Cs9g12160	−1.59	Receptor like protein 13 (RLP13), Leucine-rich repeat-containing
XLOC_023274	Cs9g12220	−2.09	Receptor like protein 13 (RLP13), Leucine-rich repeat-containing
XLOC_023264	Cs9g12040	−2.27	Receptor like protein 13 (RLP13), Leucine-rich repeat-containing
XLOC_006617	Cs3g10050	−2.30	Receptor like protein 13 (RLP13), Leucine-rich repeat-containing
XLOC_003292	–	−2.30	Receptor like protein 14 (RLP14), Leucine-rich repeat-containing
XLOC_026146	orange1.1 t02820	−1.15	Receptor like protein 15 (RLP15), Leucine-rich repeat-containing
XLOC_028464	orange1.1 t01372	−1.33	Receptor like protein 15 (RLP15), Leucine-rich repeat-containing
XLOC_025415	orange1.1 t01415	−1.66	Receptor like protein 15 (RLP15), Leucine-rich repeat-containing
XLOC_006615	Cs3g10010	−2.08	Receptor like protein 15 (RLP15), Leucine-rich repeat-containing
XLOC_015519	Cs6g12110	−2.13	Receptor like protein 15 (RLP15), Leucine-rich repeat-containing
XLOC_023261	Cs9g11990	−2.55	Receptor like protein 15 (RLP15), Leucine-rich repeat-containing
XLOC_030349	orange1.1 t05075	−1.31	Receptor like protein 22 (RLP22), Leucine-rich repeat-containing
XLOC_013590	Cs5g13820	−1.00	Receptor like protein 33 (RLP33), Leucine-rich repeat-containing
XLOC_005928	Cs2g30850	−1.63	Receptor like protein 35 (RLP35), Leucine-rich repeat-containing
XLOC_001914	Cs1g11900	−2.04	Receptor like protein 54 (RLP54), Leucine-rich repeat-containing
XLOC_030467	orange1.1 t05273	−1.52	Receptor like protein 56 (RLP56), Leucine-rich repeat-containing
XLOC_030282	orange1.1 t04923	−2.60	Receptor like protein 56 (RLP56), Leucine-rich repeat-containing
XLOC_030151	orange1.1 t06047	−1.12	Receptor like protein 6 (RLP6), Leucine-rich repeat-containing
XLOC_006431	Cs3g06220	−1.20	Receptor like protein 6 (RLP6), Leucine-rich repeat-containing
XLOC_005373	Cs2g19490	−1.17	Receptor like protein 7 (RLP7), Leucine-rich repeat-containing
XLOC_031340	–	−2.93	Receptor like protein 9 (RLP9), Leucine-rich repeat-containing
XLOC_006131	–	−1.52	Receptor-like protein kinase family protein, Leucine-rich repeat-containing
XLOC_012876	Cs5g34310	−1.09	Receptor-like protein kinase family protein, Leucine-rich repeat-containing
XLOC_015528	Cs6g12270	−2.24	Receptor-like protein kinase family protein, Leucine-rich repeat-containing
XLOC_002765	–	−2.43	Receptor-like protein kinase family protein, Leucine-rich repeat-containing
XLOC_026533	orange1.1 t03518	−1.22	Disease resistance protein (TIR-NBS-LRR class) with transmembrane receptor activity
XLOC_029900	orange1.1 t04292	−1.30	Disease resistance protein (TIR-NBS-LRR class) with transmembrane receptor activity
XLOC_014032	Cs5g22400	−1.40	Disease resistance protein (TIR-NBS-LRR class) with transmembrane receptor activity
XLOC_006408	Cs3g05870	−1.14	Disease resistance protein (CC-NBS-LRR class) family
XLOC_006396	Cs3g05690	1.99	Disease resistance protein (TIR-NBS-LRR class) with transmembrane receptor activity
XLOC_029612	orange1.1 t03700	1.14	Disease resistance protein (TIR-NBS-LRR class) with transmembrane receptor activity
XLOC_006400	Cs3g05760	1.09	Disease resistance protein (TIR-NBS-LRR class) with transmembrane receptor activity
XLOC_014130	Cs5g24240	1.08	Disease resistance protein (TIR-NBS-LRR class) with transmembrane receptor activity
Transcription factor encoding genes			
XLOC_013026	Cs5g03010	1.08	WRKY transcription factor family protein (WRKY22)
XLOC_017469	Cs7g06330	1.17	WRKY transcription factor family protein (WRKY18)
XLOC_016450	Cs6g10120	1.19	WRKY transcription factor family protein (WRKY54)
XLOC_019872	Cs7g23080	1.46	MYB transcription factor family protein
XLOC_020617	Cs8g02740	1.34	MYB transcription factor family protein
XLOC_016421	Cs6g09420	−2.27	WRKY transcription factor family protein (WRKY 4)
XLOC_028008	orange1.1 t00472	−1.26	WRKY transcription factor family protein (WRKY53)
XLOC_013000	Cs5g02450	−1.04	WRKY transcription factor family protein (WRKY53)
XLOC_005212	Cs2g16510	−2.28	MYB transcription factor family protein
XLOC_014277	Cs5g27440	−1.82	MYB transcription factor family protein
XLOC_024133	Cs9g10480	−1.54	MYB transcription factor family protein
XLOC_011371	Cs5g04290	−1.52	Homeobox transcription factor family protein
XLOC_013039	Cs5g03250	−1.29	Homeobox transcription factor family protein
XLOC_021224	Cs8g14700	−1.66	NAC domain transcription factor family protein
XLOC_021532	Cs8g21030	−1.08	NAC domain transcription factor family protein
XLOC_025849	orange1.1 t0226	−1.32	RNA-binding (RRM/RBD/RNP motifs) family protein

Among the 32 genes upregulated by DSF/Rpf-mediated *Xac* infection, three genes encode WRKY transcription factors, including WRKY18, WRKY22, and WRKY54 (Table 8). Interestingly, in *Arabidopsis*, AtWRKY18 alone with AtWRKY40 and AtWRKY60, act as negative regulators of defense signaling [59]. Other upregulated genes include two genes coding for the MYB transcription factors, three genes for the PR family proteins including one PR-5 and two PR-6, four genes for the NB-LRR family receptors, five genes for wound-responsive or –induced proteins, and a few others for disease resistance responsive proteins and stress responsive proteins (Additional file 6: Table S6).

### Expression of citrus genes associated with plant secondary metabolism and cell wall modification were altered by DSF/Rpf-mediated *Xac* infection

A total of 14 citrus genes related to the biosynthesis of flavonols, anthocyaninins, glucosinolates and terpenoids, which are well characterized defensive compounds [60], were downregulated by DSF/Rpf-mediated *Xac* infection (Additional file 7: Table S7). Five genes involved in lignin biosynthesis were upregulated by DSF/Rpf-mediated *Xac* infection, suggesting that lignin might be deposited in infected tissues, possibly as part of citrus responses to limit the pathogen colonization. Indeed, *Xac* infection induced the expression of genes related to lignin biosynthesis [61]; and, histological analyses revealed an increased lignin deposition and the existence of cell wall reinforcement in *Xac* infected tissues [62]. Remarkably, 12 genes encoding cell-wall-modifying enzymes, including expansins, endoglucanases, glycosyl transferases, and xyloglucan endotransglycosylases/hydrolases, were upregulated by DSF/Rpf-mediated *Xac* infection (Additional file 8: Table S8). Nine genes encoding protein products involved in the synthesis of cell wall precursors were also upregulated. These results implied a more pronounced effect on cell wall modification upon infection by the wild type *Xac* compared to the ∆*rpfF* mutant to limit the pathogen colonization.

## Discussion

### The *in planta* DSF/Rpf- mediated QS regulon of *Xac*

**Fig. 6:**
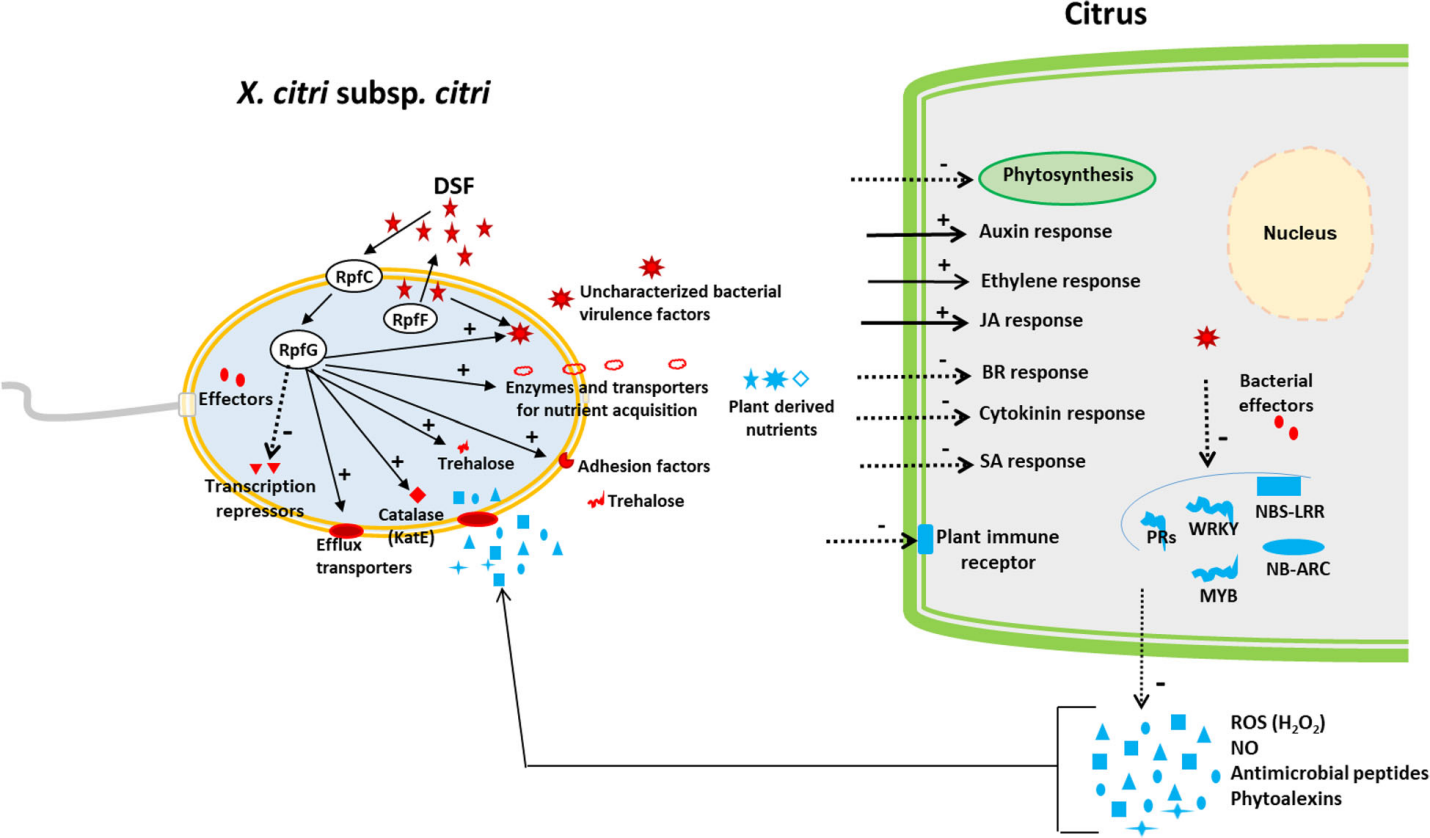
Hypothetical model of the modulation of citrus - *Xanthomonas citri* subsp. *citri* interactions by the DSF/Rpf- mediated quorum sensing (QS) during early stages of infection. Representative proteins and metabolic processes with important roles in plant-pathogen interactions are shown. Plant and bacterial molecules are depicted in light blue and red, respectively. DSF/Rpf- mediated QS modulates expression of diverse bacterial traits including adhesion, nutrition acquisition, stress tolerance (catalase and trehalose), signal transduction, transcription, and virulence factors, which collectively promote bacterial adaptation to the host environment to favor infection. The transcriptional alterations of citrus in response to DSF-mediated *X. citri* subsp. *citri* infection are characterized by the downregulation of photosynthesis, plant immune receptor-like proteins or receptor-like kinases including NBS-LRRs, NB-ARC resistance proteins, MYB and WRKY transcription factors, and pathogenesis-related (PR) proteins. Changes of phytohormone metabolism and signaling were also triggered by DSF-mediated *X. citri* subsp. *citri* infection, probably leading to increased accumulation of auxin, ethylene and jasmonic acid (JA), and decreased accumulation of brassinosteroid (BR), cytokinin and salicylic acid (SA), which may benefit the pathogen. Solid arrows with plus symbols indicate positive regulation and dashed arrows with minus symbols indicate negative regulation. Solid lines indicate information flow

The results indicate that the DSF deficiency altered *in planta* expression of 202 genes in *Xac*, with a remarkable downregulation of different sets of genes functionally involved in stress tolerance, nutrition uptake and metabolisms, signal transduction, transcriptional regulation, and virulence. These findings support the hypothesis that the DSF/Rpf- mediated QS in *Xac* modulates diverse pathogenesis traits to promote bacterial adaptation to the host environment for a successful infection (Fig. 6). For example, *Xac* cells have to counteract environmental stresses and plant generated- oxidative stress during infection on citrus host [48, 63]. Our results showed that DSF/Rpf-mediated QS contributes to stress tolerance of *Xac* by positively regulating the expression of catalase, drug resistance translocase, defense protein-degrading endoproteinase, and the MFS drug transporter (Table 1). These enzymes are collectively important for bacterial resistance against diverse stresses from the environment and/or host organisms and thus for a successful infection [38, 64, 65]. DSF/Rpf**-**mediated QS also positively regulates the biosynthesis of trehalose, which protects *Xac* cells from osmotic and oxidative stresses to enable bacterial colonization in host plants [40], i.e., in the apoplast, an osmotic stressful environment [66].

The plant apoplast is low in nitrogen and rich in plant-derived sugars such as fructose [67]. *Xac* has adapted to the apoplast with diverse nutrient acquisition strategies evolved, including diverse enzymes for plant cell wall degradation, amino acid metabolism, carbohydrate metabolism and transportation [63]. The findings in this study indicate that *Xac* exploits the DSF/Rpf -mediated QS to regulate nutrition utilization during host infection (Table 2; Table 3). Interesting, the DSF/Rpf -mediated QS positively regulates the expression of phosphate transporter encoding genes, the homologues of which in *X. axonopodis* pv*. glycines*, the causal agent of bacterial pustule of soybean, have been demonstrated to be strongly expressed at early stages of infection and required for bacterial growth in host plants to promote disease [68]. DSF/Rpf-mediated QS also regulates ferric iron uptake of *Xac in planta* (Table 3). It has been reported that Xanthoferrin, a α-hydroxycarboxylate-type siderophore produced by *Xcc* is required for its optimum virulence [69]; and, DSF positively regulates the functions involved in ferric iron uptake to promote *in planta* growth of *X. oryzae* pv. *oryzicola* [70]. However, there is no evidence that iron is limited or available to *Xac* cells grown *in planta*. The functional role of DSF/Rpf regulated ferric iron uptake in *Xac* biology and pathogenesis remains to be determined.

Importantly, the DSF/Rpf-mediated QS differentially regulated the expression of 12 determined or putative signal transducers and/or transcriptional regulators, most of which were downregulated, including the NtrB/C two-component system (Table 4). The NtrB/C system interacts with the RpfC/G system in responding to DSF signal to regulate *sigma54*-dependent promoters in *Xac* in vitro [44]. Our findings suggested that the DSF signal negatively regulates *sigma54*-dependent promoters through the RpfCG- NtrBC- *sigma54* pathway in *Xac* during early stages of host infection. The functional roles of the other signal transducers and/or transcriptional regulators regulated by the DSF/Rpf-mediated QS remain unknown. Collectively, the results suggested that DSF-mediated signaling might be linked with diverse regulators to enable complex patterns of gene expression to be employed by *Xac* to favor infection in host plants, which deserves further investigations.

Comparison of the *in planta* and in vitro DSF/RpfF regulons revealed that a set of 31 genes were commonly differentially regulated by DSF/RpfF under the two environment conditions. There are a large number of unique genes in the *in planta* regulon that were not regulated by DSF/RpfF in vitro (Additional file 5: Table S5). A couple of reasons could explain the differences among the *in planta* and in vitro DSF/RpfF regulons. It could be because of the difference in cell density of *Xac* in the two experimental conditions: approximately 10^8^ CFU/cm^2^ of leaf tissues for *in planta* experiments (Fig. 1b) and 10^9^ to 10^10^ CFU/ml of growth medium for in vitro experiments [17], as the QS regulates expression of genes in a cell density- dependent manner. It also could be because that the DSF/Rpf – mediated QS might play divers roles in regulating gene expression of *Xac* under different environment conditions. Several subsets of unique genes within the *in planta* regulon that were downregulated are involved in cell surface adhesion, stress tolerance, carbohydrate transport and metabolism, amino acids uptake and metabolism, signal transduction, and transcriptional regulation, which are in agreement with the findings produced in analysis of DSF/Rpf in vitro regulon [17]. The regulation pattern of *Xac in planta* compared to in vitro indicates the needs for real-time and in situ studies.

### Citrus transcriptional responses to DSF/Rpf-mediated *Xac* infection

Gene expression data indicated that significant transcriptional alterations occurred in citrus plants in response to DSF/Rpf-mediated *Xac* infection, which caused various changes in plant immunity and physiology, thus favoring the pathogen infection. Especially, a large group of genes differentially expressed, related to plant hormone biosynthesis, transportation, metabolism, and associated signal transduction (Table 7). The results suggested the existence of elevated levels of auxin in wild type *Xac* infected leaves compared with the ∆*rpfF* mutant infected leaves. Auxin has been shown to promote citrus canker development [71]; and auxin pathways play a role in tomato bacterial wilt caused by *Ralstonia solanacearum* [72]. Therefore, it is likely that the alterations in expression of auxin biosynthesis, mobilization and signaling genes in response to the DSF/Rpf-mediated *Xac* infection are associated with the citrus canker disease development. Additionally, cytokinin biosynthesis genes were downregulated and cytokinin metabolic genes were upregulated, implying decreased accumulation of cytokinin in wild type *Xac* infected leaves. Cytokinin has been shown to regulate plant defense responses in a dosage-dependent manner: strong activation of cytokinin signaling confers resistance to biotrophic pathogens via increased SA accumulation; by contrast, weak activation of cytokinin signaling suppresses pathogen-associated molecular pattern (PAMP)-triggered immunity (PTI) [73]. Our results suggested that the DSF/Rpf-mediated *Xac* infection modulates cytokinin accumulation and thus avoids strong activation of cytokinin signaling to promote host susceptibility. Another interesting finding is the upregulation of genes involved in the biosynthesis of and response to ethylene in wild type *Xac* infected leaves. *Xac* infection activates ethylene biosynthesis and signaling in citrus plants [61]. Ethylene is usually involved in plant defense responses against necrotrophic pathogens [74], thus it is possible that the successful establishment of *Xac* infection is favored by the development of inadequate plant defenses.

Notably, genes for JA biosynthesis and for SA production (i.e., the MeSA esterase) were upregulated in wild type *Xac* infected leaves, while genes for SA metabolic process and for BR biosynthesis or responses were downregulated (Table 7). Earlier reports showed that certain antagonistic relationships occur between BR and JA, JA and SA pathways, and BR signaling negatively regulates plant defense against pathogens [55, 75, 76]. Both biotrophic and hemibitrophic pathogens employ the antagonism between JA and SA pathways and activate JA signaling to promote infection [77, 78]. The findings in this study implied that the DSF/Rpf-mediated *Xac* infection may activate the JA signaling pathway and repress BR signaling to benefit the pathogen during early stages of infection.

Gene expression levels point to that the activity of DSF/Rpf-mediated QS might induce plant basal defenses and repress secondary defenses of citrus to promote *Xac* infection (Table 8; Additional file 6: Table S6; Additional file 7: Table S7; Additional file 8: Table S8). Remarkably, many plant immune receptor -like proteins or receptor-like kinases proteins were downregulated by DSF/Rpf-mediated *Xac* infection (Table 8), which are believed to perceive extracellular molecules, including microbe/pathogen-associated molecular patterns (M/PAMP) and environmental stimuli to induce plant basal resistance [79, 80]. In addition, four putative NB-LRR family proteins were also downregulated by DSF/Rpf-mediated *Xac* infection, which are intracellular proteins and recognize pathogen effectors to lead to strong resistance responses [81]. Overall, it is important to note that more defense- related genes were downregulated than upregulated by DSF/Rpf-mediated *Xac* infection (70 downregulated versus 32 upregulated) (Additional file 6: Table S6), especially in the group of immune receptors (34 downregulated versus 4 upregulated) (Table 8).

It is not clear how the activity of DSF/Rpf-mediated QS triggers plant basal defenses and represses secondary defenses of citrus plants. One possible explanation might lie in the observations that the DSF signal molecule itself could elicit plant defense response in *Xanthomonas*–host plant interactions and wild-type *Xanthomonas* spp. can suppress the DSF-induced defense responses by the production of the EPS xanthan and T3SS effectors [18]. Our results showed that the DSF/Rpf-mediated QS did not regulate or affect the production of the EPS xanthan by *Xac* in citrus during early stages of infection, but negatively regulated the expression of a putative T3SS effector (XAC3085) (Table 5). The homologue of XAC3085 in *X. campestris* pv. *vesicatoria* (also termed *X. euvesicatoria*), the causal agent of bacterial spot disease on pepper and tomato, was determined as a T3SS effector named XopK, whose function remains unknown but seems not to contribute to the virulence of the pathogen [82]. *Xac* might suppress the DSF molecule elicited plant defense responses through the EPS and/or the T3SS effectors that are not affected by the DSF/Rpf-mediated QS during host infection. Another possible reason might be the functional interplay between the bacterial T2SS and T3SS in modulating plant defense responses and promoting disease as observed in the *X*. *oryzae* pv. *oryzae* – rice interactions, where the bacterial T2SS secreted virulence factors: the ClsA cellulase and CbsA cellobiosidase, induced innate rice defense responses that were suppressed by T3S effectors [83]. We found that the DSF/Rpf-mediated QS positively regulates the expression of the homologue (*engXCA*/XAC0612) of the ClsA cellulase (Table 2). Therefore, wild-type *Xac* may suppress, in a T3SS-dependent manner, the citrus plant defense responses probably induced by the T2SS effector cellulase (*engXCA*/XAC0612) to enable successful infection.

## Conclusions

In conclusion, this work provides an in-depth transcriptomic analysis of DSF/Rpf -mediated QS regulation from both pathogen and host sides during the biotrophic interactions between *Xac* and citrus. Based on the results obtained, a model was presented that describes the major molecular and physiological aspects regulated by the DSF/Rpf- mediated QS during early stages of infection (Fig. 6). The findings support the hypothesis that the DSF/Rpf- mediated QS in *Xac* modulates diverse pathogenesis traits to promote bacterial adaptation to the host environment, and triggers various changes in plant immunity and physiology favoring the pathogen for successful infection. Taken together, the present work has provided novel insights into the role of the DSF/Rpf- mediated QS regulatory system in the pathogenic interactions between *Xanthomonas* and its host plants and expanded our current knowledge of DSF- mediated QS regulation, and adds to our general understanding of plant-pathogen interactions.

## Additional Files

Additional file 1: **Table S1.** Primers used for qRT-PCR assays for experimental validation (DOCX 18 kb)

Additional file 2: **Table S2.** Summary of the RNA-seq data (DOCX 13 kb)

Additional file 3: **Table S3.** Detail of the DEGs of *Xanthomonas citri* subsp*. citri* regulated by DSF/RpfF –mediated QS (DOCX 35 kb)

Additional file 4: **Table S4.** Detail of the DEGs of citrus in response to DSF/RpfF –mediated *Xac* infection (XLSX 183 kb)

Additional file 5: **Table S5.** Comparison of the in vitro and *in planta* DSF/Rpf-mediated QS regulons of *Xanthomonas citri* subsp*. citri (XLSX 29 kb)*

Additional file 6: **Table S6.** Differentially expressed citrus genes related to plant defense responses (XLSX 17 kb)

Additional file 7: **Table S7.** Differentially expressed citrus genes involved in plant secondary metabolisms (XLSX 11 kb)

Additional file 8: **Table S8.** Differentially expressed citrus genes involved in cell wall modifications (XLSX 13 kb)

## Abbreviations

bp: Base pair
cDNA: Complementary DNA
COG: Clusters of Orthologous Groups
DEGs: Differentially expressed genes
DNA: Deoxyribonucleic acid
DSF: Diffusible signal factor
EPS: Extracellular polysaccharides
FDR: False rate discovery
FPKM: Fragments per kilobase of exon permillion mapped reads
kb: Kilobases
log2FC: Log of fold change in base 2
LPS: Lipopolysaccharides
min: Minute
mM: Millimolar
mRNA: Messenger RNA
PCR: Polymerase chain reaction
pH: Hydrogenionic potential
qRT-PCR: Quantitative reverse transcription PCR
QS: Quorum sensing
RNA: Ribonucleic acid
RNA-Seq: RNA sequencing
Rpf: Regulation of pathogenicity factorsL
rpm: Rotations per minute
T2SS: Type II secretion system
T3SS: Type III secretion system
*Xac*: *X*.* citri *subsp. *citri*
*Xcc*: *X. campestris* pv. *campestris*
μg: Micrograms
μM: Micromolar
