# Supplementary material for: Diffusible signal factor (DSF)-mediated quorum sensing modulates expression of diverse traits in Xanthomonas citri and responses of citrus plants to promote disease
Source: BMC Genomics. 2019 Jan 17;20:55. doi: 10.1186/s12864-018-5384-4 (PMC6337780; doi:10.1186/s12864-018-5384-4)
Supplement: Supplementary file 1 — Table S1. Primers used for qRT-PCR assays for experimental validation (DOCX 18 kb) [file 12864_2018_5384_MOESM1_ESM.docx]

**Table S1. Primers used for qRT-PCR assays for experimental validation**

| **ID** | **Gene/Locus** | **Forward primer (5’ to 3’)** | **Reverse primer (5’ to 3’)** | **Amplicon (bp)** |
| --- | --- | --- | --- | --- |
| **For bacteria validation** | | | | |
| XAC0204 | *glnA* | GAAGAGAAGCTGATCCCGC | TGGTGACTTCCTGCATCTTC | 148 |
| XAC0205 | *glnB* | ACTTCCTGCCCAAGATCAAG | TCACGAAGATCTTGCCGTC | 120 |
| XAC0206 | *amtB* | ATCGGCAATCTGGACAAGG | AACACGTATTCCGGCAGG | 95 |
| XAC0296 | */* | CCAAGCACATCACCTCGATC | ACCAGCGCAGATAATCCATC | 138 |
| XAC0297 | */* | GATTTCCGTTCGTGATCTGC | GTGAACGATCTTGCCGATTTC | 131 |
| XAC0299 | */* | GTGCAGTTCGTGGTCAATTAC | CCGTATTCGTACAGGCTTTCC | 149 |
| XAC0300 | */* | TGTTGCTCACCGAAATCCTG | GAGGTGTCGCCGTGTAC | 148 |
| XAC0301 | *amaB* | GAGTGGAACCTGGACATCG | ATCCTTCCGCTTCCAATACC | 112 |
| XAC0310 | *vanB* | ACAAGGCTTTACGCATGGG | TGAAGTGGTCCATGAACGC | 146 |
| XAC0311 | *vanA* | CTTCAATGCCGACTACCTGAG | AAGTGGATGGTGAGCGTG | 135 |
| XAC0575 | */* | TGATCGACTTCCATTACAGCG | GCCGTTGTCCTTGAGATATTTG | 146 |
| XAC1008 | */* | TCGCTGTGTTTGAGTACGAC | CCCACAAACCGCTATCCAG | 109 |
| XAC1310 | *btuB* | TGATCAACGGCCTGGATTAC | GAATAGTTCGAGTAGACGCCG | 139 |
| XAC1397 | */* | TTGGGTTACTGCCTTTCTGG | CAAAGGAAGTTTCAGCCGTG | 148 |
| XAC1633 | *gcd* | GCGGTTTGATCTTCATTGCC | CCACCGTATTCGTACACCATC | 133 |
| XAC1793 | *celD* | AATCAGTACATCAACGCCTCC | TGCCCTTGAAGTCGTTCTG | 124 |
| XAC1794 | *sglT* | AGGGCTTTCAGTTCATCCAG | AGGCCAAGCATGAAGATCAC | 73 |
| XAC1813 | *hmsH* | AGCTGGATCTTTTCGGTAGC | AGCTATGGCGTGGATTGAAG | 129 |
| XAC1827 | */* | GCGTTTCTGCGTGACCTATG | TCAGCTCGTGGATTTCGC | 112 |
| XAC1828 | *hisG* | ATGCTGATCCGCTGGTG | ACCGTCAACACCATCAAGC | 139 |
| XAC1829 | *hisD* | GAACATCTGATTCTTGCGCTG | TGTAATCGCCGAGAAACACC | 85 |
| XAC1879 | *rpfF* | AAACCTGATCCGCCAGC | GACTTTTCATTGAGCGCCAG | 147 |
| XAC2156 | */* | TGGAAGACGGACAAAACACG | CGCTACTCGCCAACAGG | 84 |
| XAC2763 | */* | TTCCGTTGACCATCGAACTG | CCAGAATGCCGTTATCCAGTG | 150 |
| XAC2992 | *argC* | CAATACCAATTCGCCCAAACG | CCGCATCGTAGAACCAGTATG | 104 |
| XAC3451 | *ilvC* | TGAAGTTGATCGTGGACCTG | GCTGACATAATCGCCGTATTG | 92 |
| XAC3471 | *dctA* | TTCACCATCGGCAAGTACG | AGTACCGAAAACCCGCATAG | 134 |
| XAC3484 | *oprO* | ACGTCCGGAAAACGATCTG | TGAATTCGCTCTGCCACG | 137 |
| XAC3485 | *citM* | CCTATCTTTTGGTCGGGTTGG | CAACAGCGAAATCAACACCG | 86 |
| XAC3486 | *fabG* | CATTCGCTGTTCAACATCACC | GCAGTGGCGAAATTGATCTG | 128 |
| XAC3487 | *cebR* | GCGATGACCAATTACCAGAGG | TATCGAATTCCAGCCCGTG | 81 |
| XAC3507 | *celS* | GACTACAACCTCTACGGCTATC | GCCTGCGCTGTAATTGAATTTG | 135 |
| XAC4326 | *uahA* | GCAAAGCAGGGAATTTTCGG | GATGTCCCAATATCCGGTACG | 145 |
| XAC4327 | *uahA* | CCCAGCATCTACACCATTGAG | GCTAAAGTCGAGTAGGTTCACG | 99 |
| XAC4349 | */* | ACTGGGAGCTGATGTTCAC | ACGCGATTGAGCAGATGG | 76 |
| XAC0612 | *engXCA* | ATCTGGGACCGCCATTTC | TCCTTCGTTGATGCCCTTG | 150 |
| XAC1579 | *oprO* | CATCAAGTACAAGTTTGGCGG | GAGATGCCATAGGTGTTCGTC | 144 |
| XAC1632 | */* | GACTCTTTCACTGACACGGG | ATCCAGAATCGTCATCCGTTG | 95 |
| XAC3513 | */* | GTAGCTCGGTTCCGCAG | TGTGTTCGATGACGATGCG | 147 |
| XAC2151 | *yapH* | TGGAATCTGCTTACCAACGG | GGTACTGAAGGTGTCGCTG | 138 |
| **For plant validation** | | | | |
| XLOC_000150 | Cs1g03980 | TCCCCTGTTTTCATTCCCG | ACAAAAGAACTCCCCAAGCC | 120 |
| XLOC_013855 | Cs5g18930 | TGTCTCACCATGTGTTAGCAG | GTGGAATGTAGGAGAAACTGGG | 149 |
| XLOC_001696 | Cs1g07950 | CAAACGAAATCCTTGGCACC | GCATTACGGTAGCTGTTGAGAG | 142 |
| XLOC_011371 | Cs5g04290 | TCCTTCTCCTTCTCCTTTTCTTG | AGTCCTCATCCCCATTTTCATC | 128 |
| XLOC_014277 | Cs5g27440 | GATCTTGAGCCCTTGTCCTATG | AACATCCTCAACTTCCTCGC | 142 |
| XLOC_013000 | Cs5g02450 | GCGAAGCGATAAACACAAGTG | TTAGTCTACATGGGCGGTTTG | 133 |
| XLOC_016421 | Cs6g09420 | CACCAACGAGTAATCCCATAGG | AATTCTTGGCTCCCTCACAG | 125 |
| XLOC_017469 | Cs7g06330 | CTCCTGTTCCAAATGCCAAG | CCGAGGTGAGGGATTATCTTTAG | 134 |
| XLOC_001537 | Cs1g05240 | CTTTGGAGTTGTGTTGCTGG | GATCAACCTCTTCACCCTCTAC | 149 |
| XLOC_020268 | Cs7g30900 | CATCTCCGAGAATCCATGGTC | GCCTCTTCCTCTTCAAAACCG | 132 |
| XLOC_019229 | Cs7g09590 | GGCAAACCCAGTACTATACCAG | AGCCAGACATACAAAATACCCG | 147 |
| XLOC_001450 | Cs1g03280 | AGCCCTAACTCTGATTCAATCC | AACGAAATCCACTAGCCTGAG | 148 |
| XLOC_003353 | Cs2g09980 | GTGATTTTGGTGTTGGCGG | CAGCCTTTTCGAACTCAAAGC | 113 |
| XLOC_029132 | orange1.1t02723 | ACTGGGTTTATTGAGCGGG | CCATCACCATTTGTATCAGCATC | 137 |
| XLOC_013935 | Cs5g20600 | CCCACAGTTCAATTTTCACCG | CCACGAATACCCATAGATACCAG | 95 |
| XLOC_003682 | Cs2g16560 | TCTTTGCCTCTGATGTCCTTC | GACTCCCCAGAACAGCATG | 82 |
| XLOC_026677 | orange1.1t03773 | TCTTTTGGTTAAGGGACGAGG | CAGGATCAAGTGTGCTTTTCAG | 108 |
| XLOC_009232 | Cs4g03220 | CTTGGAACTTTGATGGTTGCC | GAGGCTTTATCAAGGGACAGAG | 125 |
| XLOC_029032 | orange1.1t02529 | CAAAATTACTGCCCTGACAACG | TCTGTTGCCGATGTTCCATG | 101 |
| XLOC_019001 | Cs7g05180 | ACCTACCAACGAGATGCAAC | TTATCCCACAAGCCTTCACC | 88 |
| XLOC_023393 | Cs9g14450 | CATTCTTTGCTCCTGCTCTTTC | GGCGAAGTGTTTGGAGTTTTG | 119 |
| XLOC_030349 | orange1.1t05075 | AGGAGTCTCTGTTTTCAAGCG | AGAACCAATCCTGCCACAAG | 82 |
| XLOC_026600 | orange1.1t03628 | TTACAGGGTGATTTGGGCTC | CAAACTGGTTTTCCGCCATG | 147 |
| XLOC_011787 | Cs5g12930 | GGTGATGTTTGGGATACTCTTTG | GGAGCTCTGTCCGGAAATATC | 143 |
| XLOC_007213 | Cs3g21660 | CTTCATTTCCCCTGCTTTTCG | ATGACCAGAATGTGACCACG | 122 |
| XLOC_016450 | Cs6g10120 | TCAGGCCACATCTTCATCTTC | ATCGCTTTTGACCCTTCTCC | 147 |
| XLOC_002571 | Cs1g24440 | AGAGGAAGCCATGACCAAAC | TGACTGCCCAACTTATCGC | 150 |
| XLOC_024251 | Cs9g12830 | GAAATCGGCAACTTGACCATG | GCTGATACCACACCAACTAGG | 143 |
| XLOC_024252 | Cs9g12840 | CCTCCATCTACACCATGCATAC | TTCGGAAGCTCACCAGAAAG | 145 |
| XLOC_023092 | Cs9g08670 | TCAGATTGTGTTGGAAGGGAC | CGGTTCTTAGTATTGCCTCTGG | 137 |
| XLOC_023091 | Cs9g08660 | GGCATCGGAATGTAGAGGTAAG | GGATTCTCCCTCTCAACTGTTG | 90 |
| XLOC_003365 | Cs2g10140 | AATGATCTTGAGTCCCTATGGTG | CTGATGTGCCTTTGAGATGC | 147 |
| XLOC_013736 | Cs5g16770 | CTATCTTGTCCTAGACGCTGC | ATCACCAACTAGAAGCAGACG | 78 |
| **Reference gene for qRT-PCR** | | | | |
| XAC1631 | *gyrA* | CGTCACGTTGATCCGTTTGT | GCTTGCTTCGTCCACTCCCT |  |
| *CtGAPDH* |  | GGAAGGTCAAGATCGGAATCAA | CGTCCCTCTGCAAGATGACTCT |  |
