# Supplementary material for: Diffusible signal factor (DSF)-mediated quorum sensing modulates expression of diverse traits in Xanthomonas citri and responses of citrus plants to promote disease
Source: BMC Genomics. 2019 Jan 17;20:55. doi: 10.1186/s12864-018-5384-4 (PMC6337780; doi:10.1186/s12864-018-5384-4)
Supplement: Supplementary file 2 — Table S2. Summary of the RNA-seq data (DOCX 13 kb) [file 12864_2018_5384_MOESM2_ESM.docx]

**Table S2:**  **Summary of the RNA-seq data**

| **Sample ID** | **Clean reads** | **Total reads per biological condition** | **Reads mapped uniquely to Xac strain 306 (%)** | **Reads mapped uniquely to Citrus reference (%)** | **Total mapped reads (%)** |
| --- | --- | --- | --- | --- | --- |
| WT1 | 64,828,318 | 227,390,638 | 2,360,078 (3.64%) | 41,199,699 (63.55%) | 47,844,394 (73.80%) |
| WT2 | 82,329,608 |  | 4,559,782 (5.54%) | 55,925,447 (67.93%) | 65,961,636 (80.12%) |
| WT3 | 80,232,712 |  | 3,545,711 (4.42%) | 54,300,925 (67.68%) | 63,175,786 (78.74%) |
| MU1 | 79,715,132 | 278,592,424 | 1,902,036 (2.39%) | 53,810,294 (67.50) | 61,365,665 (76.98%) |
| MU2 | 81,778,336 |  | 1,948,541 (2.38%) | 55,080,292 (67.35%) | 62,977,654 (77.01%) |
| MU3 | 117,098,956 |  | 2,815,475 (2.40%) | 79,714,805 (68.07%) | 90,646,505 (77.41%) |

WT: samples from *Xanthomonas citri* subsp. *citri* wild type strain 306 inoculated plants. MU: samples from ∆*rpfF* mutant strain inoculated plants.
