# Supplementary material for: Diffusible signal factor (DSF)-mediated quorum sensing modulates expression of diverse traits in Xanthomonas citri and responses of citrus plants to promote disease
Source: BMC Genomics. 2019 Jan 17;20:55. doi: 10.1186/s12864-018-5384-4 (PMC6337780; doi:10.1186/s12864-018-5384-4)
Supplement: Supplementary file 3 — Table S3. Detail of the DEGs of Xanthomonas citri subsp. citri regulated by DSF/RpfF –mediated QS (DOCX 35 kb) [file 12864_2018_5384_MOESM3_ESM.docx]

**Table S3. Detail of the DEGs regulated by DSF/RpfF –mediated QS in *Xanthomonas citri* subsp*. citri***

| **Locus** | **Gene** | **Log_2_Fold Change**  **(Wt/**∆***rpfF*)** | **Annotation** | **COG Category** |
| --- | --- | --- | --- | --- |
| XAC0029 | *egl* | 1.343767 | cellulase | 2730G (Carbohydrate transport and metabolism) |
| XAC0151 |  | 1.005125 | hypothetical protein | - |
| XAC0159 | *estA1* | 1.148544 | carboxylesterase type B | 2272I (Lipid transport and metabolism) |
| XAC0174 | *phhA* | -1.14187 | phenylalanine 4-monooxygenase | 3186E (Amino acid transport and metabolism) |
| XAC0204 | *glnA* | -3.39413 | glutamine synthetase | 0174E (Amino acid transport and metabolism) |
| XAC0205 | *glnB* | -3.0114 | nitrogen regulatory protein P-II | 0347E (Amino acid transport and metabolism) |
| XAC0206 | *amtB* | -2.78533 | ammonium transporter | 0004P (Inorganic ion transport and metabolism) |
| XAC0207 | *ntrB* | -1.27923 | two-component system sensor protein | 3852T (Signal transduction mechanisms) |
| XAC0208 | *ntrC* | -1.20796 | two-component system regulatory protein | 2204T (Signal transduction mechanisms) |
| XAC0215 |  | -1.16991 | hypothetical protein | 2959H (Coenzyme transport and metabolism) |
| XAC0217 | *lgtB* | -1.06402 | glycosyltransferase | 2730G (Carbohydrate transport and metabolism) |
| XAC0256 | *mls* | -1.40453 | malate synthase | 2225C (Energy production and conversion) |
| XAC0257 | *aceA* | -1.59798 | isocitrate lyase | 2224C (Energy production and conversion) |
| XAC0285 |  | 1.221961 | hypothetical protein | - |
| XAC0295 |  | -1.64173 | hypothetical protein | purine nucleobase metabolic process |
| XAC0296 |  | -2.5022 | monoxygenase | 2072P (Inorganic ion transport and metabolism) |
| XAC0297 |  | -2.93576 | hypothetical protein | 3195S (Function unknown)/ allantoin biosynthetic process |
| XAC0298 |  | -1.83938 | hypothetical protein | Nuclear transport factor 2 (NTF2-like) superfamily |
| XAC0299 |  | -2.15959 | hypothetical protein | 0726G (Carbohydrate transport and metabolism) |
| XAC0300 | *pucG* | -2.08012 | serine-pyruvate aminotransferase | 0075E (Amino acid transport and metabolism) |
| XAC0301 | *amaB* | -2.72662 | allantoate amidohydrolase | 0624E (Amino acid transport and metabolism) |
| XAC0310 | *vanB* | -3.94374 | vanillate O-demethylase oxidoreductase | 4638PR (Inorganic ion transport and metabolism) |
| XAC0311 | *vanA* | -3.0752 | vanillate O-demethylase oxygenase | 4638PR (Inorganic ion transport and metabolism) |
| XAC0334 | *sflA* | 1.603409 | NADH-dependent FMN reductase | 2959H (Coenzyme transport and metabolism) |
| XAC0335 |  | 1.877482 | hypothetical protein | - |
| XAC0336 | *metE* | 1.717641 | 5-methyltetrahydropteroyltriglutamate-- | 0620E (Amino acid transport and metabolism) |
|  |  |  | homocysteine S-methyltransferase |  |
| XAC0375 | *aes* | -1.42345 | lipase | 0657I (Lipid transport and metabolism) |
| XAC0376 |  | -1.36924 | hypothetical protein | - |
| XAC0421 | *mdoB* | -1.1698 | phosphoglycerol transferase I | 1368M (Cell wall/membrane/envelope biogenesis) |
| XAC0425 | *glgA* | 1.029436 | glycogen synthase | 0297G (Carbohydrate transport and metabolism) |
| XAC0429 | *glgY* | 1.042535 | malto-oligosyltrehalose synthase | 3280G (Carbohydrate transport and metabolism) |
| XAC0465 |  | 1.370298 | metalloproteinase | 3227E (Amino acid transport and metabolism) |
| XAC0510 |  | -1.22328 | hypothetical protein | 1289S (Function unknown) |
| XAC0575 | *ganB* | -1.97928 | arabinogalactan endo-1,4-beta-galactosidase | 3867G (Carbohydrate transport and metabolism) |
| XAC0599 |  | 1.231328 | hypothetical protein | - |
| XAC0607 |  | -1.05899 | hypothetical protein | 5373S (Function unknown) |
| XAC0612 | *engXCA* | 1.528411 | cellulase | 2730G (Carbohydrate transport and metabolism) |
| XAC0654 | *acoR* | -1.27456 | transcriptional regulator AcoR | 3284QK (Secondary metabolisms, and Transcription) |
| XAC0690 | *fecA* | -1.08552 | TonB-dependent receptor | 1629P (Inorganic ion transport and metabolism) |
| XAC0742 |  | -1.45881 | hypothetical protein | 1629P (Inorganic ion transport and metabolism) |
| XAC0906 | *ahpF* | -1.01416 | alkyl hydroperoxide reductase | 3634O (Post-translational modification) |
| XAC0907 | *ahpC* | -1.13931 | alkyl hydroperoxide reductase | 0450O (Post-translational modification, chaperones) |
| XAC0928 |  | 1.114922 | extracellular protease | 1404O (Post-translational modification) |
| XAC0999 | *cirA* | -1.04769 | colicin I receptor | 1629P (Inorganic ion transport and metabolism) |
| XAC1008 |  | -2.24402 | hypothetical protein/ Cell_wall_hydrolase | 3773M (Cell wall/membrane/envelope biogenesis) |
| XAC1034 |  | 1.381218 | peptidyl-Asp metalloendopeptidase | proteolysis |
| XAC1037 |  | 1.11915 | hypothetical protein | 0671I (Lipid transport and metabolism) |
| XAC1038 | *gtrB* | 1.118426 | glycosyl transferase | 2730G (Carbohydrate transport and metabolism) |
| XAC1086 | *dniR* | -1.30705 | murein hydrolase D | 1388M (Cell wall/membrane/envelope biogenesis) |
| XAC1149 | *bfr* | 1.007015 | bacterioferritin | 2193P (Inorganic ion transport and metabolism) |
| XAC1206 |  | 1.060327 | hypothetical protein | - |
| XAC1210 |  | 1.072634 | hypothetical protein | - |
| XAC1211 | *KatE* | 1.000901 | catalase | 0376P Peroxidase I(Inorganic ion transport and metabolism) |
| XAC1214 | *gcvP* | 1.090561 | glycine dehydrogenase | 1003E (Amino acid transport and metabolism) |
| XAC1286 | *abfA* | -1.09065 | alpha-L-arabinofuranosidase | 3534G (Carbohydrate transport and metabolism) |
| XAC1308 | *bga* | -1.17747 | beta-galactosidase | 3250G (Carbohydrate transport and metabolism) |
| XAC1309 | *galA* | -1.49244 | arabinogalactan endo-1,4-beta-galactosidase | 3867G (Carbohydrate transport and metabolism) |
| XAC1310 | *btuB* | -2.06982 | TonB-dependent receptor | 1629P (Inorganic ion transport and metabolism) |
| XAC1316 | *mmsB* | 1.029047 | 3-hydroxyisobutyrate dehydrogenase | 2084I (Lipid transport and metabolism) |
| XAC1328 |  | 1.074227 | hypothetical protein/CheY-like_superfamily | signal transducer activity |
| XAC1357 | *yegD* | -1.49989 | heat shock protein | 0443O (Post-translational modification) |
| XAC1358 | *slyD* | -1.7936 | peptidyl-prolyl cis-trans isomerase | 1047O (Post-translational modification) |
| XAC1363 | *araJ* | 1.31904 | arabinose efflux permease, MFS transporter | 2814G (Carbohydrate transport and metabolism) |
| XAC1387 |  | -1.08673 | hypothetical protein | 4731S (Function unknown) |
| XAC1393 |  | -1.14578 | hypothetical protein | 4805S (Function unknown) |
| XAC1397 |  | -2.05562 | hypothetical protein | - |
| XAC1433 | *asnB* | -1.19433 | asparagine synthetase B | 0367E (Amino acid transport and metabolism) |
| XAC1466 | *pcp* | -1.1277 | peptidoglycan outer membrane lipoprotein | 3133M (Cell wall/membrane/envelope biogenesis) |
| XAC1471 |  | -1.12782 | hypothetical protein | 3133M (Cell wall/membrane/envelope biogenesis) |
| XAC1512 |  | 1.195411 | serine peptidase | 1404O (Post-translational modification) |
| XAC1556 | *fucP* | -1.43457 | glucose-galactose transporter | 0738G (Carbohydrate transport and metabolism) |
| XAC1557 | *scrK* | -1.4929 | fructokinase | 0524G (Carbohydrate transport and metabolism) |
| XAC1558 |  | -1.46347 | hypothetical protein | 2942G (Carbohydrate transport and metabolism) |
| XAC1578 | *phoX* | 1.342531 | phosphate-binding protein | 0226P (Inorganic ion transport and metabolism) |
| XAC1579 | *oprO* | 1.502927 | polyphosphate-selective porin O | 3746P (Inorganic ion transport and metabolism) |
| XAC1632 |  | 1.711967 | hypothetical protein | - |
| XAC1633 | *gcd* | 2.059043 | glucose dehydrogenase | 4993G (Carbohydrate transport and metabolism) |
| XAC1651 | *tonB* | -1.19822 | TonB-like protein | 0810M (Cell wall/membrane/envelope biogenesis) |
| XAC1768 | *fhuA* | -1.19682 | TonB-dependent receptor | 1629P (Inorganic ion transport and metabolism) |
| XAC1769 | *cirA* | -1.71538 | TonB-dependent receptor | 1629P (Inorganic ion transport and metabolism) |
| XAC1770 | *celA* | -1.02627 | cellulase | 0726G (Carbohydrate transport and metabolism) |
| XAC1771 |  | -1.01899 | sialic acid-specific 9-O-acetylesterase | 0726G (Carbohydrate transport and metabolism) |
| XAC1780 | *amiC* | -1.06001 | N-acetylmuramoyl-L-alanine amidase | 0860M (Cell wall/membrane/envelope biogenesis) |
| XAC1793 | *celD* | -2.4606 | glucan 1,4-beta-glucosidase | 1472G (Carbohydrate transport and metabolism) |
| XAC1794 | *folk* | -2.37812 | sodium/glucose cotransport protein | 0726G (Carbohydrate transport and metabolism) |
| XAC1812 | *hmsF* | -1.72231 | HmsF protein | 0726G (Carbohydrate transport and metabolism) |
| XAC1813 | *hmsH* | -2.06157 | HmsH protein | 4783R (General function prediction only) |
| XAC1820 | *thrA* | -1.24383 | bifunctional aspartokinase I | 0527E (Amino acid transport and metabolism) |
| XAC1821 | *thrB* | -1.20356 | homoserine kinase | 0083E (Amino acid transport and metabolism) |
| XAC1823 | *thrC* | -1.24202 | threonine synthase | 0498E (Amino acid transport and metabolism) |
| XAC1827 |  | -2.41471 | hypothetical protein | 4496S (Function unknown) |
| XAC1828 | *hisG* | -2.31628 | ATP phosphoribosyltransferase | 0040E (Amino acid transport and metabolism) |
| XAC1829 | *hisD* | -2.01946 | histidinol dehydrogenase | 0141E (Amino acid transport and metabolism) |
| XAC1830 | *hisC* | -1.9395 | histidinol-phosphate aminotransferase | 0079E (Amino acid transport and metabolism) |
| XAC1831 | *hisB* | -1.73897 | imidazole glycerol-phosphate dehydratase/histidinol phosphatase | 0131E (Amino acid transport and metabolism) |
| XAC1832 | *hisH* | -1.36807 | imidazole glycerol phosphate synthase subunit | 0118E (Amino acid transport and metabolism) |
| XAC1833 | *hisA* | -1.60818 | imidazole-4-carboxamide isomerase | 0106E (Amino acid transport and metabolism) |
| XAC1834 | *hisF* | -1.51311 | imidazole glycerol phosphate synthase subunit | 0107E (Amino acid transport and metabolism) |
| XAC1835 | *hisI* | -1.11676 | bifunctional phosphoribosyl-AMP cyclohydrolase/pyrophosphatase | 0139E (Amino acid transport and metabolism) |
| XAC1879 | *rpfF* | 2.268775 | enoyl-CoA hydratase | 1024I (Lipid transport and metabolism) |
| XAC1883 |  | -1.00294 | hypothetical protein | 2002K (Transcription) |
| XAC1884 |  | -1.25551 | hypothetical protein | 1487R (General function prediction only) |
| XAC1885 | *acnB* | -1.3419 | bifunctional aconitate hydratase 2/2-methylisocitrate dehydratase | 1049C (Energy production and conversion) |
| XAC1926 |  | 1.342953 | hypothetical protein | - |
| XAC1927 | *aslB* | 1.142193 | Fe-S oxidoreductase | 0641R (General function prediction only) |
| XAC2012 | *fadA* | -1.25342 | acetyl-CoA acetyltransferase | 0183I (Lipid transport and metabolism) |
| XAC2013 | *fadB* | -1.65865 | 3-hydroxyacyl-CoA dehydrogenase | 1250I (Lipid transport and metabolism) |
| XAC2014 |  | -1.28926 | TetR family transcriptional regulator | 1309K (Transcription) |
| XAC2113 |  | -1.19436 | hypothetical protein | 3115D (Cell cycle control, cell division) |
| XAC2125 | *gtrB* | 1.068551 | glycosyl transferase-like protein | 0726G (Carbohydrate transport and metabolism) |
| XAC2151 | *yapH* | 1.473502 | YapH protein/Filamentous hemagglutinin-related protein | 4625S (Function unknown) |
| XAC2155 |  | 1.357612 | hypothetical protein | 3685S (Function unknown) |
| XAC2156 |  | 1.971843 | hypothetical protein | 3729 Stress-induced protein |
| XAC2312 |  | -1.27066 | hypothetical protein/TonB-dependent outer membrane receptor | 1629P (Inorganic ion transport and metabolism) |
| XAC2411 | *acvB* | -1.07905 | virulence protein | 3946U (Intracellular trafficking, secretion, and transport) |
| XAC2494 | *yieO* | 1.293744 | drug resistance translocase | 2814G (Carbohydrate transport and metabolism) |
| XAC2501 | *fruB* | -1.73007 | multiphosphoryl transfer protein | 1080G (Carbohydrate transport and metabolism) |
| XAC2502 | *fruK* | -1.67967 | 1-phosphofructokinase | 1105G (Carbohydrate transport and metabolism) |
| XAC2503 | *fruA* | -1.79056 | PTS system fructose-specific transporter subunit IIBC | 1299G (Carbohydrate transport and metabolism) |
| XAC2504 | *rpfN* | -1.64214 | regulator of pathogenicity factors | 3659M (Cell wall/membrane/envelope biogenesis) |
| XAC2547 | *dapA* | 1.064452 | dihydrodipicolinate synthetase | 0329EM (Amino acid transport and metabolism) |
| XAC2548 |  | 1.26636 | Oxidoreductase | 0446R (General function prediction only) |
| XAC2615 | *virB3* | 1.036795 | VirB3 protein | 3702U (Intracellular trafficking, secretion, and transport) |
| XAC2663 |  | 1.097319 | transposase | - |
| XAC2746 | *pepO* | -1.45947 | metallopeptidase | 3590O (Post-translational modification) |
| XAC2755 |  | -1.0497 | hypothetical protein | 0791M (Cell wall/membrane/envelope biogenesis) |
| XAC2763 | *mep* | 2.003668 | extracellular protease | 1404O (Post-translational modification) |
| XAC2821 |  | -1.02237 | hypothetical protein | 3904S (Function unknown) |
| XAC2931 |  | -1.03312 | hypothetical protein | - |
| XAC2934 |  | -1.02389 | hypothetical protein/IscR | 1959K (Transcription) |
| XAC2935 | *ynhE* | -1.06608 | cysteine desulfurase | 0719O (Post-translational modification) |
| XAC2936 | *ynhD* | -1.13226 | ABC transporter ATP-binding protein | 0396O (Post-translational modification) |
| XAC2937 | *ynhC* | -1.13523 | ABC transporter permease | 0719O (Post-translational modification) |
| XAC2992 | *argC* | 2.98411 | endoproteinase ArgC | (Function unknown) |
| XAC3073 |  | 1.00336 | hypothetical protein; | 3858R (General function prediction only) |
| XAC3085 |  | -1.06402 | hypothetical protein | 4987CO (Energy production and conversion) |
| XAC3120 | *glk* | 1.357571 | glucokinase | 0837G (Carbohydrate transport and metabolism) |
| XAC3155 |  | -1.2657 | hypothetical protein | - |
| XAC3168 | *bfeA* | -1.54936 | ferric enterobactin receptor | 4771P (Inorganic ion transport and metabolism) |
| XAC3169 | *bfeA* | -1.17555 | ferric enterobactin receptor | 4771P (Inorganic ion transport and metabolism) |
| XAC3212 | *gcd* | 1.046132 | glucose dehydrogenase | 4993G (Carbohydrate transport and metabolism) |
| XAC3218 | *comL* | -1.00268 | competence lipoprotein | 4105R (General function prediction only) |
| XAC3300 | *estA* | -1.10366 | esterase | 3240IR (Lipid transport and metabolism, General function) |
| XAC3377 |  | -1.13481 | hypothetical protein | 0598P (Inorganic ion transport and metabolism) |
| XAC3379 | *moxR* | -1.06728 | methanol dehydrogenase regulatory protein | 0714R (General function prediction only) |
| XAC3439 |  | -1.16234 | hypothetical protein | - |
| XAC3451 | *ilvC* | -2.15548 | ketol-acid reductoisomerase | 0059EH (Coenzyme transport and metabolism) |
| XAC3452 | *ilvG* | -1.69241 | acetolactate synthase 2 catalytic subunit | 0028EH (Coenzyme transport and metabolism) |
| XAC3453 | *ilvM* | -1.49778 | acetolactate synthase isozyme II small subunit | 0028EH Coenzyme transport and metabolism) |
| XAC3454 | *tdcB* | -1.70866 | threonine dehydratase | 1171E (Amino acid transport and metabolism) |
| XAC3455 | *leuA* | -1.2211 | 2-isopropylmalate synthase | 0119E (Amino acid transport and metabolism) |
| XAC3470 | *maeB* | -1.77582 | malic enzyme | 0281C (Energy production and conversion) |
| XAC3471 | *dctA* | -2.80231 | C4-dicarboxylate transporter DctA | 1301C (Energy production and conversion) |
| XAC3472 | *oprO* | -1.82297 | polyphosphate-selective porin O | 3746P (Inorganic ion transport and metabolism) |
| XAC3474 | *cit1* | -1.07624 | citrate carrier protein | 2814G (Carbohydrate transport and metabolism) |
| XAC3482 | *tctE* | -1.02021 | two-component system sensor protein | 0642T (Signal transduction mechanisms) |
| XAC3484 | *oprO* | -2.90319 | porin | 3746P (Inorganic ion transport and metabolism) |
| XAC3485 | *citM* | -3.15219 | Mg++/citrate complex transporter | 2851C (Energy production and conversion) |
| XAC3486 | *fabG* | -3.14208 | 3-ketoacyl-ACP reductase | 1028IQR (Lipid transport and metabolism) |
| XAC3487 | *cebR* | -2.20118 | transcriptional regulator | 1609K (Transcription) |
| XAC3489 | *fyuA* | -1.49252 | TonB-dependent receptor | 1629P (Inorganic ion transport and metabolism) |
| XAC3490 |  | -1.22598 | amylosucrase or alpha amylase | 0366G (Carbohydrate transport and metabolism) |
| XAC3506 |  | -1.67582 | hypothetical protein | - |
| XAC3507 |  | -1.99962 | hypothetical protein | - |
| XAC3513 |  | 1.79506 | hypothetical protein | - |
| XAC3514 |  | 1.747712 | serine protease | - |
| XAC3525 |  | -1.11299 | hypothetical protein | - |
| XAC3533 |  | 1.227255 | hypothetical protein | 2814G [Carbohydrate transport and metabolism] |
| XAC3545 |  | 1.125448 | protease | 1404O (Post-translational modifications) |
| XAC3546 | *xadA* | 1.297434 | Outer membrane protein | - |
| XAC3664 | *OmpW* | -1.13007 | outer membrane protein | 3047M (Cell wall/membrane/envelope biogenesis) |
| XAC3703 |  | -1.02414 | hypothetical protein | 2335M (Cell wall/membrane/envelope biogenesis) |
| XAC3720 |  | -1.17712 | hypothetical protein | 3275T (Signal transduction mechanisms) |
| XAC3745 |  | 1.171205 | hypothetical protein | - |
| XAC3753 |  | -1.22625 | hypothetical protein | - |
| XAC3754 |  | -1.00178 | hypothetical protein | - |
| XAC3755 |  | -1.05341 | hypothetical protein | 2885M (Cell wall/membrane/envelope biogenesis) |
| XAC3760 |  | -1.00474 | hypothetical protein | 2866E (Amino acid transport and metabolism) |
| XAC3769 | *nucA* | 1.490817 | endonuclease | 1864F (Nucleotide transport and metabolism) |
| XAC3777 |  | 1.089779 | hypothetical protein | - |
| XAC3797 |  | -1.01908 | hypothetical protein | 3307M (Cell wall/membrane/envelope biogenesis) |
| XAC3856 |  | -1.1914 | hypothetical protein | - |
| XAC3921 | *ugt* | 1.517816 | glucosyltransferase | 1819GC (Carbohydrate transport and metabolismn) |
| XAC3922 | *entF* | 1.418815 | ATP-dependent serine activating enzyme | 1020Q (Secondary metabolisms, transport) |
| XAC3927 |  | 1.04117 | hypothetical protein | 3642T (Signal transduction mechanisms) |
| XAC3957 |  | 1.045283 | hypothetical protein | - |
| XAC3959 |  | -1.68547 | hypothetical protein | 3239I (Lipid transport and metabolism) |
| XAC3960 |  | -1.51141 | oxidoreductase | 1018C (Energy production and conversion) |
| XAC3966 |  | 1.391852 | hypothetical protein | 3133M (Cell wall/membrane/envelope biogenesis) |
| XAC4024 |  | -1.06229 | hypothetical protein | 4775M (Cell wall/membrane/envelope biogenesis) |
| XAC4048 | *iroN* | -1.62516 | TonB-dependent receptor | 4206H (Coenzyme transport and metabolism) |
| XAC4167 |  | 1.287797 | hypothetical protein | 3540P (Inorganic ion transport and metabolism) |
| XAC4182 |  | 1.126833 | cytochrome C biogenesis protein | 1225O (Post-translational modification) |
| XAC4195 | *ndvB/celAP* | -1.23098 | NdvB protein/ cellobionic acid phosphorylase | 3459G (Carbohydrate transport and metabolism) |
| XAC4219 |  | -1.09076 | hypothetical protein | 2930S (Function unknown) |
| XAC4256 | *cirA* | -1.32958 | TonB-dependent receptor | 1629P (Inorganic ion transport and metabolism) |
| XAC4259 | *blc* | 1.05027 | Lipocalin | 3040M (Cell wall/membrane/envelope biogenesis) |
| XAC4326 | *uahA* | 6.50375 | urea amidolyase | 4770I (Lipid transport and metabolism) |
| XAC4327 | *uahA* | 5.924104 | allophanate hydrolase | 0139E Amino acid metabolism |
| XAC4349 |  | -1.96983 | Oxireductase | 0604CR (Energy production and conversion) |
| XAC4355 |  | -1.3438 | hypothetical protein/Glyco_hydro like | 3537G (Carbohydrate transport and metabolism) |
| XAC4361 | *ttuB* | -1.50627 | MFS transporter | 2271G (Carbohydrate transport and metabolism) |
| XACb0046 | *virB3* | 1.015197 | VirB3 protein | 3702U (Intracellular trafficking, secretion, and transport) |
